# Reactive Oxygen Species, Antioxidant Agents, and DNA Damage in Developing Maize Mitochondria and Plastids

**DOI:** 10.3389/fpls.2020.00596

**Published:** 2020-05-19

**Authors:** Diwaker Tripathi, Andy Nam, Delene J. Oldenburg, Arnold J. Bendich

**Affiliations:** Department of Biology, University of Washington, Seattle, WA, United States

**Keywords:** maize, ROS, plastids, mitochondria, protoplasts, DNA damage

## Abstract

Maize shoot development progresses from non-pigmented meristematic cells at the base of the leaf to expanded and non-dividing green cells of the leaf blade. This transition is accompanied by the conversion of promitochondria and proplastids to their mature forms and massive fragmentation of both mitochondrial DNA (mtDNA) and plastid DNA (ptDNA), collectively termed organellar DNA (orgDNA). We measured developmental changes in reactive oxygen species (ROS), which at high concentrations can lead to oxidative stress and DNA damage, as well as antioxidant agents and oxidative damage in orgDNA. Our plants were grown under normal, non-stressful conditions. Nonetheless, we found more oxidative damage in orgDNA from leaf than stalk tissues and higher levels of hydrogen peroxide, superoxide, and superoxide dismutase in leaf than stalk tissues and in light-grown compared to dark-grown leaves. In both mitochondria and plastids, activities of the antioxidant enzyme peroxidase were higher in stalk than in leaves and in dark-grown than light-grown leaves. In protoplasts, the amount of the small-molecule antioxidants, glutathione and ascorbic acid, and catalase activity were also higher in the stalk than in leaf tissue. The data suggest that the degree of oxidative stress in the organelles is lower in stalk than leaf and lower in dark than light growth conditions. We speculate that the damaged/fragmented orgDNA in leaves (but not the basal meristem) results from ROS signaling to the nucleus to stop delivering DNA repair proteins to mature organelles producing large amounts of ROS.

## Introduction

Reactive oxygen species (ROS) can have both detrimental and beneficial effects on plants. At high concentrations, ROS can lead to oxidative stress by causing damage to various biomolecules. ROS are produced as unavoidable byproducts of electron transport reactions in both respiration and photosynthesis, and damage-defense measures are employed to ameliorate oxidative stress. But ROS also function in modifying the cell wall during development, as signaling molecules to maintain cellular and organismal homeostasis, and to regulate plant development (Mittler, 2017; Smirnoff and Arnaud, 2019). For example, ROS can affect the distribution of chloroplasts within a cell and the ability to resist pathogen attack (Park et al., 2018; Ding et al., 2019), as well as the fate of stem cells (Zeng et al., 2017; Yang et al., 2018).

Reactive oxygen species can be produced in chloroplasts, mitochondria, and several other plant cell compartments (Janku et al., 2019). Since ROS are produced during the partial reduction of molecular oxygen, one way to avoid the potential damaging effects of ROS is to maintain certain cells under hypoxic conditions. In mammals, a hypoxic niche is maintained during early development in cells that will develop into gametes (embryonic stem cells) and later in the stem cells that provide the differentiated cells of adult tissues (Mohyeldin et al., 2010). Similarly, the non-green cells of the shoot apical meristem in Arabidopsis are also maintained in a hypoxic niche for 5 weeks during the development of the inflorescence meristem of the adult plant (Weits et al., 2019). Thus, in both animals and plants, the DNA that will be transmitted to the next generation is protected from potential oxidative stress associated with respiration and photosynthesis (Mohyeldin et al., 2010; Considine et al., 2017).

Although the nucleus is not a major site of ROS production, ROS signaling molecules generated elsewhere in the cell can be moved to the nucleus to modulate expression of nuclear genes (Noctor and Foyer, 2016). The highest concentration of ROS should be found in the parts of the cell that produce most of the ROS, such as chloroplasts and mitochondria, but most research on DNA damage has focused on nuclear DNA. The most severe type of DNA damage is a double-strand break, and both homologous recombination (HR) and non-homologous end-joining (NHEJ) are used to repair this break in the nuclear DNA of yeasts and mammals (Runge and Li, 2018; Scully et al., 2019). Both HR and NHEJ are also found in the plant nucleus (Spampinato, 2017), but thus far only HR has been identified in either mitochondria or plastids of plants (Boesch et al., 2011; Christensen, 2018). Other DNA damage repair systems found in the nucleus may also be found in mitochondria and plastids. For example, Arabidopsis organellar DNA (orgDNA) polymerases can perform microhomology-mediated end-joining (MMEJ) *in vitro* (Garcia-Medel et al., 2019), proteins associated with base excision repair (BER) have been found in Arabidopsis plastids and mitochondria (Gutman and Niyogi, 2009; Boesch et al., 2011), and the BER system is the major pathway for repair of oxidatively damaged DNA (Markkanen, 2017).

As maize plants develop from the meristem at the base of the shoot (the basal meristem) to the leaves, the size of orgDNA [referring to both plastid DNA (ptDNA) and mitochondrial DNA (mtDNA)] decreases from molecules equal to or greater than the size of the genome (570 kb for mtDNA and 140 kb for ptDNA) in the meristem to much smaller fragments in the leaf (Oldenburg and Bendich, 2004, 2015; Kumar et al., 2014). In dark-grown plants, high-integrity ptDNA (large complex-branched molecules) is retained in the leaves, and a rapid decline in ptDNA copy number is observed after transfer from dark to light growth conditions (Zheng et al., 2011). We interpreted this decline in molecular integrity to ROS-induced DNA damage that was not repaired, followed by degradation of the unrepaired orgDNA molecules (Oldenburg and Bendich, 2015).

Through the decades, research has focused on the damage caused by ROS. More recently, however, the positive aspects of ROS signaling have been appreciated when oxidative stress is increased by environmental change (extreme temperature, intense light, high salinity, water or nutrient deprivation) or deleterious mutations (Halliwell, 2006; Noctor and Foyer, 2016; Brunkard and Burch-Smith, 2018; Foyer, 2018; Bokhari and Sharma, 2019; Zandalinas et al., 2019). Our approach has been to monitor changes in orgDNA during the normal development of the wild-type plant, without the imposition of genotoxic agents or extreme environments. In particular, we wish to investigate potential ROS signaling that leads to the demise of orgDNA in differentiated somatic cells but not in germline cells.

Here, we report on the types and levels of ROS in mitochondria, plastids, and whole cells during maize seedling development in light and dark growth conditions so as to assess the correlation between ROS and orgDNA degradation. We also report on antioxidant agents and oxidative damage to orgDNA as assessed by levels of 8-hydroxydeoxyguanasine (8-OHdG) and 8-oxoguanine (8-oxoG).

## Materials and Methods

### Plant Tissue

*Zea mays* (inbred line B73) seeds were imbibed overnight and sown in Sunshine soil Mix #4 and vermiculite (1:1 ratio). The seedlings were grown for 12 days with a 16 h light/8 h dark photoperiod (light-grown) or in continuous dark for 12 days (dark-grown). The light intensity was ∼500 μmol s^–1^ m^–2^. Seedlings were washed with 0.5% sarkosyl for ∼3 min and then rinsed with distilled water. For each assay, tissue was harvested from 20 to 25 plants as follows: Stalk lower (base of stalk 5 mm above the node); Stalk upper (top of stalk 5 mm below the ligule of the first leaf), leaf blades (L1 or L1 + L2 + L3). Stalk tissue was composed of several concentric rings of leaves, the outermost being the first leaf sheath. L1 was the fully expanded blade, whereas L2 and L3 were still developing. The coleoptile was removed before the extraction of plastids, mitochondria, and protoplasts.

### Isolation of Plastids and Mitochondria

Plastids and mitochondria were isolated using high-salt buffer (HSB; 1.25 M NaCl, 40 mM HEPES pH 7.6, 2 mM EDTA pH 8, 0.1% BSA) (Oldenburg et al., 2006, 2013). 0.1% β-mercaptoethanol (Sigma-Aldrich) was added to the buffer before grinding the tissue samples. Briefly, leaf and stalk tissues were homogenized in HSB using a blender, and the homogenate was filtered through 1–3 layers of Miracloth (EMD Millipore). The homogenate was differentially centrifuged first at low speed (500 × *g* for 5 min) to remove nuclei. Then the supernatant was centrifuged (3,000 × *g* for 10 min) to pellet plastids. The resulting supernatant was centrifuged at 20,000 × *g* for 15 min) to pellet mitochondria. The plastid and mitochondria pellets were washed three times with chloroplast dilution buffer (CDB; 0.33 M D-sorbitol, 20 mM HEPES pH 7.6, 2 mM EDTA, 1 mM MgCl_2_, 0.1% BSA) and mitochondria dilution buffer (MDB; 0.4 M D-sorbitol, 0.1 M HEPES pH 7.6, 2 mM EDTA, 1 mM MgCl_2_, 0.1% BSA), respectively. The plastids and mitochondria were further purified using discontinuous (step) Percoll gradients as follows. For plastids, 30% and 70% Percoll solutions adjusted to the equivalent osmolarity of 1x CDB were prepared (for example: for 30%, 12 mL Percoll + 8 mL 5x Chlp Gradient Buffer + 20 mL dH_2_O and for 70%, 28 mL Percoll + 8 mL 5x Chlp Gradient Buffer + 4 mL dH_2_O; 5x Chlp Gradient Buffer is 1.65 M D-sorbitol, 40 mM Hepes pH 7.6, 4 mM EDTA, 2 mM MgCl_2_, 0.2% BSA). For two-step gradients, 15 mL 30% Percoll was layered onto 15 mL of 70% Percoll in a 40-mL centrifuge tube. Then 2–4 mL of plastid solution was gently layered on top, followed by centrifuged for 30 min at 1,500 × *g* using a JA-20 fixed-angle rotor. Plastids were removed from the 30/70 Percoll interface, transferred to a centrifuge tube and washed 2–3 times with CDB (using 10x the volume of recovered plastid solution), followed by centrifugation of 3,000 × *g* for 8 min to pellet plastids. The purified plastids were then resuspended in a small volume of CDB. A similar process was used for purification of mitochondria, except for the following minor changes. A two-step 28% and 45% Percoll gradient, with solutions adjusted to the equivalent osmolarity of 1x MDB, was used (for example: for 28%, 11.2 mL Percoll + 20 mL 2x Mito Gradient Buffer + 8.8 mL dH_2_O and for 45%, 18 mL Percoll + 20 mL 2x Mito Gradient Buffer + 2 mL dH_2_O; 2x Mito Gradient Buffer is 0.8 M D-sorbitol, 40 mM Hepes pH 7.6, 4 mM EDTA, 2 mM MgCl_2_, 0.2% BSA). Centrifugation was done for 20 min at 20,000 × *g* using a JA-20 fixed-angle rotor, mitochondria were recovered from the 28/45 Percoll interface, washed 2–3 times with MDB, pelleted by centrifugation for 15 min at 20,000 × *g*, and resuspended in small volume of MDB. Finally, plastids and mitochondria were stored in CDB or MDB. Freshly isolated plastids and mitochondria were used in the ROS assays.

### Isolation of DNA From Organelles

Plastid and mtDNA were extracted using cetyltrimethylammonium bromide (CTAB) as described by Rogers and Bendich (1985) with minor modifications. An equal volume of 2x CTAB buffer [2% CTAB (w/v), 100 mM Tris/HCl (pH 8.0), 20 mM EDTA, 1.4 M NaCl, 1% polyvinylpyrrolidone (M 40000; w/v); preheated to 65°C] and Proteinase K (20 μg/ml) were added to the resuspended plastids or mitochondria and incubated at 65°C for 1 h. Then 0.1 M phenylmethylsulfonyl fluoride was added, followed by incubation at room temperature for 1 h. Then RNase A was added to 100 μg/mL, and the samples were kept at 60°C for 15 min. Next, potassium acetate was added to 400 mM, and the mixtures were kept on ice for 15 min before centrifugation at 12,000 × *g* for 10 min at 4°C. Equal volumes of chloroform:isoamyl alcohol (24:1) were added, the tubes were shaken, and then centrifuged at 12,000 × *g* for 1 min. After isopropanol precipitation, the DNA pellet was suspended in 10 mM Tris (pH 8), 1 mM EDTA (TE), and precipitated with two volumes of 100% ethanol overnight at −20°C before pelleting. DNA pellets were washed three times with 70% ethanol, dried, and then resuspended in TE. Quantitation was performed using the Quant-IT DNA quantitation kit (Thermo Fisher Scientific).

### Protoplast Isolation

Maize protoplasts were isolated from the leaf and stalk tissues, as described by Sheen (1995). Briefly, seedlings were washed with 0.5% Sarkosyl (5 min), 0.6% sodium hypochlorite (10 min), and 70% ethanol (10 s) and rinsed with sterile water. 0.5 mm strips were cut from the middle part of four or five leaves and stalks. Tissues were digested in the enzyme solution (1.5% cellulase R10 and 0.3% macerozyme (Yakult Honsha) in 0.6 M mannitol, 10 mM MES pH 5.7, 1 mM CaCl_2_, 5mM β-mercaptoethanol, 0.1% BSA for 30 min in a vacuum and 2 h at room temperature with agitation at 80 rpm. Protoplasts were stored overnight at 4°C. The suspension containing protoplasts was filtered through a 35 μm nylon mesh. Protoplasts were pelleted by centrifuging at 150 × *g*, and the pellet was washed twice and then stored in W1 buffer (154 mM NaCl, 125 mM CaCl_2_, 5 mM KCl, 2 mM MES pH 5.7). Protoplasts were counted using a counting chamber slide. Freshly isolated protoplasts were used for the assays.

### Assays of ROS and Antioxidant Agents

#### ROS Marker Dyes

Fluorescein and rhodamine dyes are chemically reduced to colorless, non-fluorescent dyes. These “dihydro” derivatives are readily oxidized back to the parent dye by ROS and thus can serve as fluorogenic probes for detecting oxidative activity in cells and tissues. The fluorogenic probes and CellROX Green reagent (Thermo Fisher Scientific) were used to measure ROS in plastids, and the rhodamine dye DHR123 (Thermo Fisher Scientific) was used to measure ROS in mitochondria and protoplasts. Equal volumes of centrifuged pellets of isolated plastids, mitochondria, and protoplasts from leaf/stalk were used for each comparative measurement. Resuspended plastids/mitochondria/protoplasts were incubated with 5 μM CellROX or DHR123 for 30 min at 37°C before the fluorescence units were measured using a Victor plate reader (Perkin Elmer) at 485/520 nm (for CellROX) and 507/529 nm (for DHR123).

For superoxide detection, MitoSOX Red (Thermo Fisher Scientific) was used as a mitochondrial superoxide indicator. Oxidation of MitoSOX Red indicator (or dihydroethidium) by superoxide results in the formation of 2-hydroxyethidium that exhibits fluorescence at 510/580 nm.

#### Amplex Red Assays for H_2_O_2_ and Peroxidase Activity

For measuring H_2_O_2_ and peroxidase levels, we used the Amplex red dye. Amplex red reagent (10-acetyl-3,7-dihydroxyphenoxazine from Thermo Fisher Scientific) reacts with H_2_O_2_ in a 1:1 stoichiometry to produce highly fluorescent resorufin that can be measured by absorbance at 560 nm. The manufacturer’s protocol was followed to measure H_2_O_2_ and peroxidase. Briefly, H_2_O_2_ and peroxidase standards were prepared by serial dilution. Equal volumes (as above) of plastids/mitochondria/protoplasts and H_2_O_2_/peroxidase standard solutions were added to the Amplex red reagent and incubated at room temperature for 30 min. The absorbance was measured, and H_2_O_2_ and peroxidase levels were calculated using standard curves.

#### SOD Activity Assay

Superoxide dismutase (SOD) activity was measured using a SOD colorimetric activity kit and the manufacturer’s protocol (Thermo Fisher Scientific). This assay measures all types of SOD activity, including Cu/Zn, Mn, and FeSOD types. Samples (plastids/mitochondria/protoplasts) were diluted in colored sample diluent and added to the wells of a 96-well plate. The substrate was added followed by Xanthine Oxidase Reagent and incubation at room temperature for 20 min. Superoxide is generated by the xanthine oxidase that converts a colorless substrate to a yellow-colored product, which was quantified at 450 nm by an absorbance assay. A SOD standard curve was used for all samples.

#### Catalase Assay

Catalase activity in protoplasts was quantified using the OxiSelect^TM^ catalase activity assay according to the manufacturer’s protocol (Cell Biolabs) that involves decomposition of H_2_O_2_ into water and oxygen, which is proportional to the concentration of catalase. After the reaction, the catalase is quenched with sodium azide, and the remaining H_2_O_2_ facilitates the coupling reaction of 4-aminophenazone (4-aminoantipyrene, AAP) and 3,5-dichloro-2-hydroxy-benzenesulfonic acid (DHBS) in the presence of horseradish peroxidase (HRP) catalyst. The product, quinoneimine dye, was measured at 520 nm using a 96-well microtiter plate. Hydrogen peroxide ‘working solution’ was added to each well. Incubation was at room temperature for 40–60 min with vigorous mixing. The absorbance was read at 520 nm. The activity in the samples was determined by interpolation of the catalase standard curve.

#### Ascorbic Acid Assays

The levels of ascorbic acid (AsA) in protoplasts were quantified using the Cell Biolabs’ OxiSelect^TM^ Ascorbic Acid Assay kit according to the manufacturer’s protocol (Cell Biolabs). The assay was based on the Ferric Reducing/Antioxidant Ascorbic Acid (FRASC) chemistry driven by the electron-donating reducing power of antioxidants. The assay employs ascorbate oxidase, which allows the user to differentiate the AsA content from other antioxidants present within the samples. AsA levels in a sample are determined by measuring the difference in optical density between two sample wells, one with and one without the enzyme. In samples, the ferrous iron was chelated to a colorimetric probe to form a product that was measured at 540. AsA levels (nM) were determined using the standard curve.

#### Glutathione Assays

Total glutathione (GSH) levels in protoplasts were determined by the OxiSelect^TM^ Total Glutathione Assay Kit as per the manufacturer’s protocol (Cell Biolabs). In this assay, GSH reductase reduces oxidized glutathione (GSSG) to reduced glutathione (GSH) in the presence of NADPH. Subsequently, the chromogen reacts with the thiol group of GSH to produce a colored compound that absorbs at 405 nm. The total glutathione content (μM) was determined by comparison with the predetermined GSH standard curve.

#### DNA Damage Assays

##### ELISA 8-OHdG assay

The quantitative measurement of 8-OHdG was determined by the OxiSelect^TM^ Oxidative DNA Damage ELISA kit (Cell Biolabs) following the manufacturer’s protocol, which includes digestion of DNA to nucleosides. Equal amounts of the digested ptDNA and mtDNA and 8-OHdG standards were added to wells of 8-OHdG/BSA-conjugate preabsorbed microwell strips and incubated at room temperature for 10 min on an orbital shaker. Then anti-8-OHdG antibody was added to each well, and the plate was incubated at room temperature for 1 h. The strips were washed with Wash Buffer three times, and the diluted secondary antibody-enzyme conjugate was added for incubation at room temperature for 1 h. After washing, a substrate solution was added, and incubation was at room temperature for 2 min. The absorbance of each microwell was measured using 450 nm, and the 8-OHdG level was measured using a standard curve.

##### 8-OxoG immunofluorescence assay

Plastids and mitochondria from light-grown stalk lower, stalk upper and L1 tissues were fixed in 4% formaldehyde/Dulbecco’s phosphate buffered saline (PBS; Gibco) in 1 mM EDTA for 10 min, pelleted, and washed twice in PBS/EDTA. Then organelles were permeabilized in 0.1% Triton X-100/PBS/EDTA for 5 min, followed by washing twice. Fixed and permeabilized plastids were incubated in blocking solution (2% BSA/PBS/EDTA) for 30 min. Organelles were incubated with primary antibody anti-8-oxoguanine mouse monoclonal (1:1000 dilution; 1 μL of 0.5 mg/mL anti-8-oxoG in 1 mL 1% BSA/PBS/EDTA blocking solution) (Millipore-Sigma MAB3560-C) for 1 h at room temperature, then washed three times. Next, organelles were incubated with secondary antibody goat anti-mouse IgM conjugated with Alexa Fluor 488 (1:1000 dilution; 1 μL of 2 mg/mL Alexa in 1 mL 1% BSA/PBS/EDTA blocking solution) (Invitrogen A21042) for 1 h at room temperature, then washed three times.

For plastids, immunofluorescence imaging was done using a Nikon Microphot Epifluorescence microscope and images acquired with a QImaging Retiga 1300 10-bit digital camera using OpenLab image capture and analysis software. Imaging of 8-oxoG/Alexa 488 plastids was done using a 470/40ex and 525/50em filter set and autofluorescence imaging of plastids with a 470/20ex and 514em filter set. For individual plastids, the 8-oxoG/Alexa 488 mean fluorescence intensity (FI) was measured as pixel values (0 to 1023) (background mean FI was also measured and subtracted from plastid mean FI). As a control, plastids incubated with the Alexa secondary Ab and without the 8-oxoG primary antibody were also imaged. Immunofluorescence analysis of 8-oxoG/Alexa was also performed with plastids from light- and dark-grown entire seedling shoots (stalk and leaves), and the fluorescence intensity was evaluated visually (“by eye”) using a scale of undetectable, weak, or high fluorescence.

The mitochondria were stained with MitoTracker Red CMXRos (Invitrogen/Molecular Probes) (incubation in 1x PBS/EDTA/200 nM CMXRos for 1 h) prior to fixation. Imaging of mitochondria with the MitoTracker dye and 8-oxoG/Alexa 488 was done using an Olympus IX81 microscope and images were acquired with a Hamamatsu Orca Flash 2.8 CMOS 12-bit digital camera. A TRITC filter set (556/20 ex and 614/30 em) was used for the MitoTracker dye and a FITC filter set (485/20 ex and 516/11 em) for 8-oxoG/Alexa 488. For individual mitochondria, the 8-oxoG/Alexa 488 mean fluorescence intensity (FI) was measured as pixel values (0 to 4096) (background mean FI was also measured and subtracted from mitochondria mean FI). As a control, mitochondria incubated with the Alexa secondary Ab and without the 8-oxoG primary antibody were also imaged. Differences in fluorescence intensity, higher for mitochondria (Figure 6B) than plastids (Figure 6A), can be attributed to the use of two different systems for image acquisition, the Hamamatsu 12-bit and the QImaging 10-bit, respectively.

### Statistical Analysis

**FIGURE 1:**
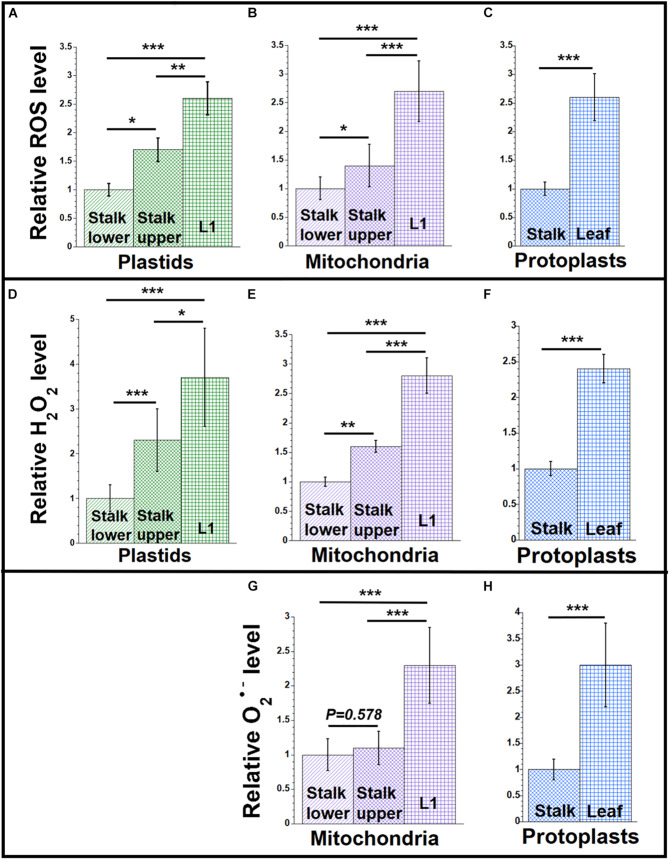
ROS levels during maize development. Plastids and mitochondria were isolated from light-grown maize seedling tissues (Stalk lower, basal 1/3 of stalk; Stalk upper, upper 2/3 of stalk; L1, leaf 1). Protoplasts were isolated from Leaf (combined leaves L1, L2, and L3) and the entire Stalk (stalk lower and upper). For each set of assays, equal volumes of plastids, mitochondria, or protoplasts were used. Measurements are given relative to the tissue with the lowest value (stalk lower or stalk), which is set at one (see the section “Materials and Methods”). **(A–C)** The level of reactive oxygen species (ROS; superoxide anion (O_2_^•–^), hydroxyl radical (HO^•^), and H_2_O_2_) was measured using the oxidative stress marker fluorescence dyes DHR123 for mitochondria and protoplasts and CellROX green for plastids. **(D–F)** H_2_O_2_ in was measured using the Amplex red assay. The hydrogen peroxide concentrations in μM were measured. **(G,H)** Superoxide anion was measured using the mitochondrial-specific superoxide fluorescence dye, MitoSOX. All assays were performed at least three times. Statistically significant differences were measured using ANOVA statistic test with *post hoc* analysis using Tukey’s HSD and are shown as asterisks, where *P-value ≤ 0.05, **P-value ≤ 0.01, ***P-value ≤ 0.001. *P*-values > 0.05 are indicated on respective graphs.

**FIGURE 2:**
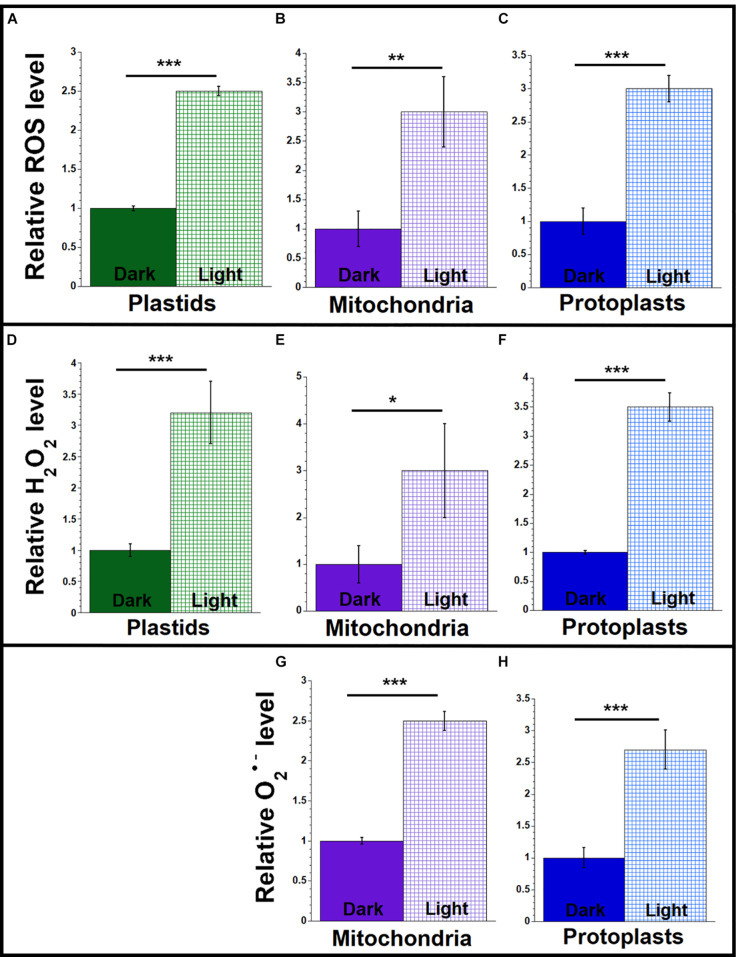
ROS levels in light and dark conditions. Plastids, mitochondria, and protoplasts were isolated from light-grown (light) and dark-grown (dark) maize seedling leaves (L1 + L2 + L3). Equal volumes of plastids, mitochondria or protoplasts were used for each set of assays. The assay measurements are given relative to the tissue with the lowest value (dark-grown samples) which is set at one. **(A–C)** The levels of ROS (DHR123 for plastids and mitochondria and CellROX for protoplasts) were assayed as in Figure 1. **(D–F)** The level of H_2_O_2_ was measured using the Amplex red as in Figure 1. **(G,H)** The levels of superoxide anion (O_2_^•–^) were measured using MitoSOX as in Figure 1. All assays were performed at least three times. Statistically significant differences were measured using ANOVA statistic test with *post hoc* analysis using Tukey’s HSD and are shown as asterisks, where *P-value ≤ 0.05, **P-value ≤ 0.01, ***P-value ≤ 0.001.

**FIGURE 3:**
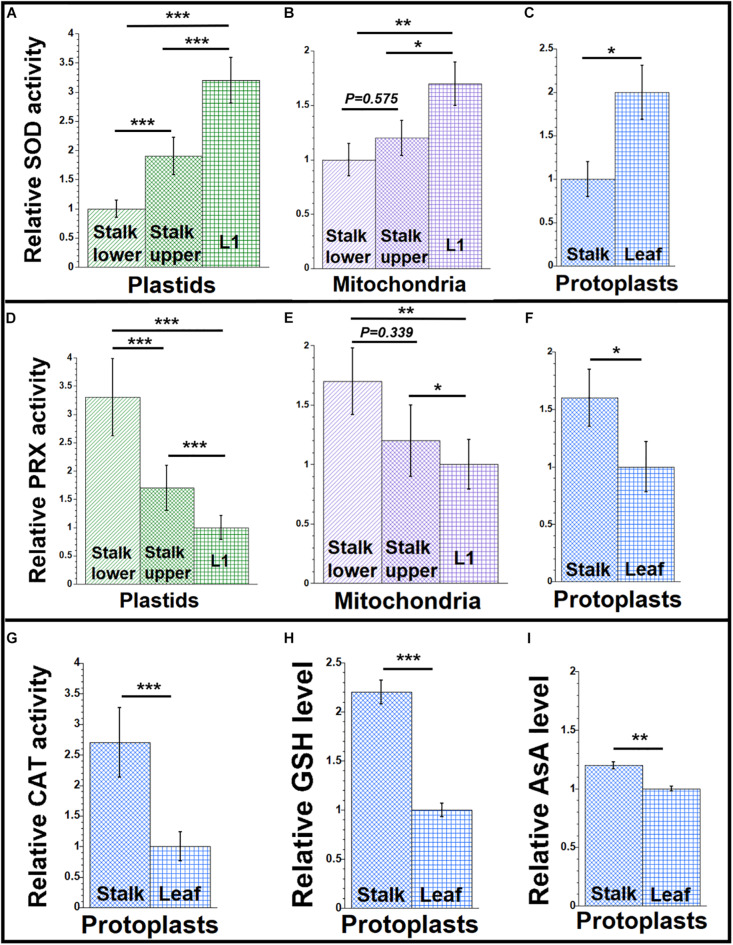
Antioxidant agents during maize development. Organelles and protoplasts were isolated, as in Figure 1. The assay measurements are given relative to the tissue with the lowest value which is set at one. **(A–C)** The level of superoxide dismutase (SOD) enzyme activity was measured as U/mL using an immunoassay. **(D–F)** The Amplex red assay was used for the peroxidase (PRX) enzyme activity and measured as mU/mL. **(G)** A colorimetric assay was used for determining catalase (CAT) enzyme activity and measured as U/mL. **(H)** Total glutathione (GSH) and **(I)** ascorbic acid (AsA) levels (μM and nM, respectively) in protoplasts were determined by colorimetric assays. All assays were performed at least three times. Statistically significant differences were measured using ANOVA statistic test with *post hoc* analysis using Tukey’s HSD and are shown as asterisks, where *P-value ≤ 0.05, **P-value ≤ 0.01, ***P-value ≤ 0.001. *P*-values > 0.05 are indicated in the respective graphs.

**FIGURE 4:**
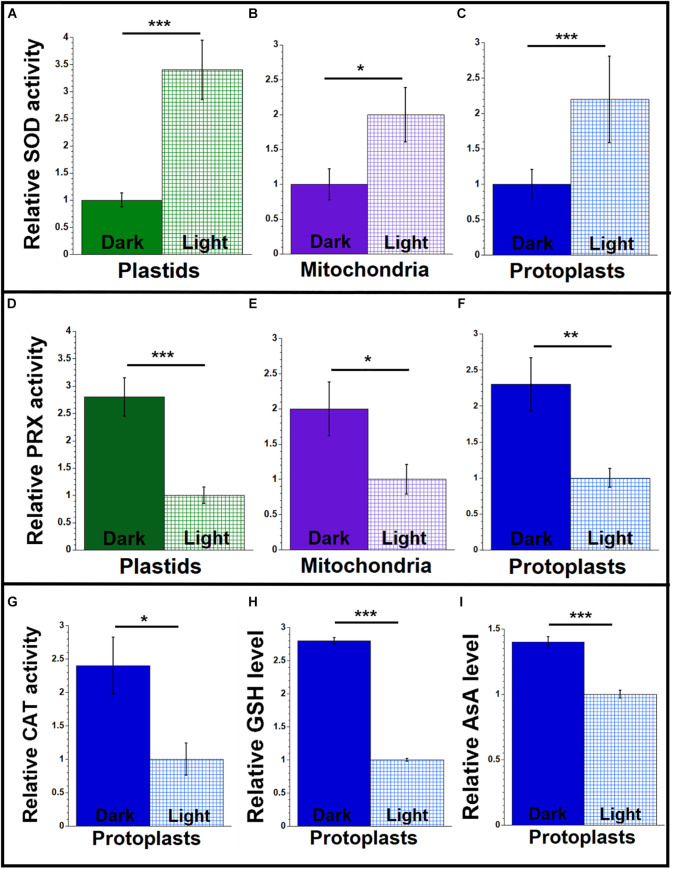
Antioxidant agents from maize seedlings grown under light or dark conditions. Organelles and protoplasts were isolated, as in Figure 2. The assay measurements are given relative to the tissue with the lowest value, which is set at one. **(A–C)** The level of superoxide dismutase (SOD) enzyme activity was measured as U/mL using an immunoassay. **(D–F)** The level of peroxidase (PRX) enzyme activity was measured as mU/mL using the Amplex red assay. **(G)** A colorimetric assay was used for determining catalase (CAT) enzyme activity and measured as U/mL. **(H)** Total glutathione (GSH) and **(I)** ascorbic acid (AsA) levels (μM and nM, respectively) in protoplasts were determined by colorimetric assays. All assays were performed at least three times. Statistically significant differences were measured using ANOVA statistic test with *post hoc* analysis using Tukey’s HSD and are shown as asterisks, where *P-value ≤ 0.05, **P-value ≤ 0.01, ***P-value ≤ 0.001.

**FIGURE 5:**
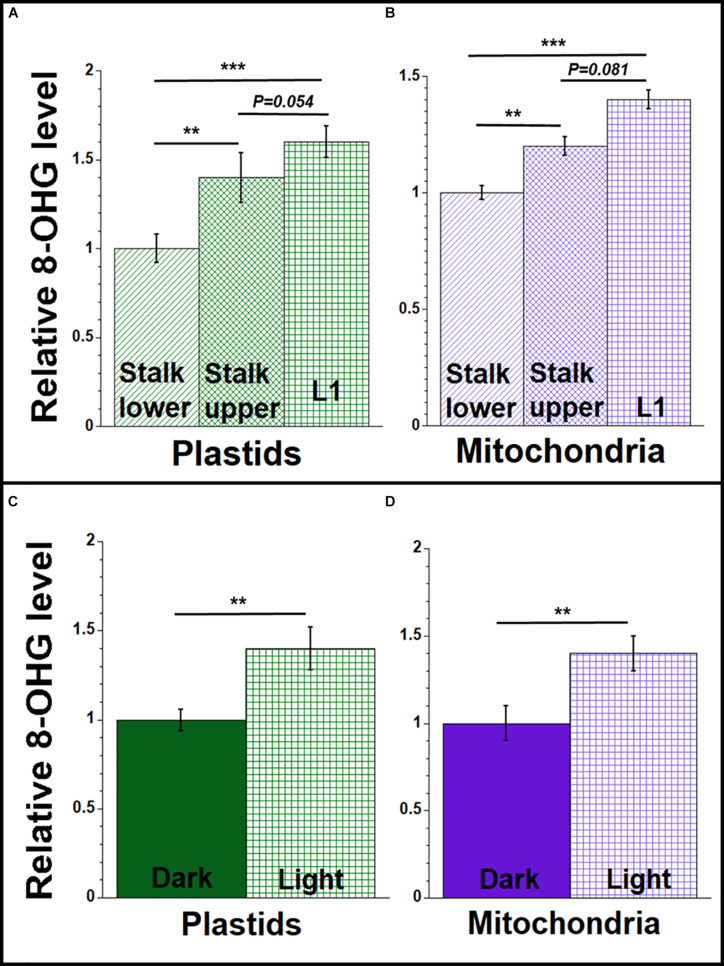
8-hydroxydeoxyguanosine (8-OHdG) representing orgDNA damage during maize development. Equal amounts of DNA extracted from isolated plastids and mitochondria were used to measure the 8-OHdG levels using a competitive ELISA assay. The levels of 8-OHdG in ptDNA and mtDNA were determined (ng/mL) and shown relative to stalk lower for **(A,B)** and relative to dark-grown leaves for **(C,D)**. All assays were performed at least three times. Statistically significant differences were measured using ANOVA statistic test with *post hoc* analysis using Tukey’s HSD and are shown as asterisks, where *P-value ≤ 0.05, **P-value ≤ 0.01, ***P-value ≤ 0.001. *P*-values > 0.05 are indicated on respective graphs.

All assays were performed at least three times with similar results. For Figures 1–5, the values in each bar graph are shown as mean relative values ± SE from three independent assays (biological replicates). Statistically significant differences between tissues were assessed by the Student’s *t*-test and/or by the ANOVA, and Tukey honest significant difference test and are shown as asterisks, where *^∗^P*-value ≤ 0.05, *^∗∗^P*-value ≤ 0.01, *^∗∗∗^P*-value ≤ 0.001, and *P*-values > 0.05 are indicated on respective graphs (see Supplementary Material).

## Results

We previously reported changes in the structure of orgDNA molecules during maize development and proposed that light triggers orgDNA degradation probably due to ROS-induced damage without subsequent repair (Oldenburg and Bendich, 2004; Oldenburg et al., 2006, 2013; Zheng et al., 2011; Kumar et al., 2014). There is a gradient in cell and organellar development from the base of the stalk to the tip of the maize leaf (Sylvester et al., 1990; Stern et al., 2004). Here, we measured the levels of ROS, antioxidant agents, and orgDNA damage at three stages of maize development: stalk lower (the base of the stalk), stalk upper (top of the stalk), and the blades from the first three leaves (Table 1 and see “Materials and Methods” section). L1 refers to the first and oldest leaf. L2 and L3 refers to the second and third leaves, respectively. The tissue with the lowest value in each set of assays was used as the baseline for comparison with other tissues and is set at 1 (see “Materials and Methods” section and see Supplementary Material for statistical analyses).

**TABLE 1:** Assays for ROS and antioxidant agents.

**Assay**	**Method**	**Mechanism**	**Detection**
General ROS	Dihydrorhodamine 123 and CellROX dyes	Dyes fluoresce green upon oxidation	Fluorescence
Hydrogen peroxide	Amplex red assay (Amplex red reagent + horseradish peroxidase)	Generation of fluorescent resorufin	Absorbance/fluorescence
Superoxide	MitoSOX dye	Dye oxidized by superoxide in mitochondria	Fluorescence
Superoxide dismutase	Colorimetric immunoassay	Generation of a yellow product	Absorbance
Peroxidase	Amplex red assay (Amplex red reagent + hydrogen peroxide)	Generation of fluorescent resorufin	Absorbance/fluorescence
Catalase	Colorimetric assay	Coupling of quinoneimine dye with H_2_O_2_	Absorbance
Glutathione	Colorimetric assay	Generation of a colored compound by a reaction of chromogen with glutathione	Absorbance
Ascorbic acid	Colorimetric assay	Ferric reducing/antioxidant ascorbic acid (FRASC) chemistry	Absorbance

In order to compare properties of orgDNA molecules during maize development, we previously used equal volumes of isolated packed organelles, and here we use the same standard to assess changes in ROS and orgDNA damage. Organelle number per cell, organelle size, and protein amount and composition per organelle all change greatly during the transition from promitochondria and proplastids to mature organelles. Therefore, neither an equal number of organelles nor an equal amount of protein is a good standard for comparison. How changes in ROS assessed using isolated organelles might reflect changes in the organelles within the plant will be considered later (see “Discussion” section).

### ROS Levels Increase During Development

To measure the ROS level, we used ROS-indicator dyes that quantify general ROS components or that are specific for either superoxide anion (O_2_^•–^) or hydrogen peroxide (H_2_O_2_). We used these dyes (see “Materials and Methods” section) with isolated plastids and mitochondria and with whole-cell protoplasts. The ROS-indicator dyes are oxidized to fluorescent products that were quantified using a microplate reader.

Figure 1 shows relative ROS levels using the dyes DHR123 for mitochondria and protoplasts and CellROX for plastids (Supplementary Table S1). Figure 1A shows that the ROS level is lowest in the plastids isolated from the stalk lower tissue. As the developmental gradient proceeds from stalk lower to stalk upper to the blade of L1, the ROS level increases to 2.6 in L1 compared to stalk lower. Unless accompanied by a corresponding increase in antioxidant defense, we would expect greater ROS damage to molecules of ptDNA in the green leaf blade than in the stalk (see “Discussion” section). A similar developmental increase of 2.7-fold was found for ROS in mitochondria isolated from the same tissues (Figure 1B), as well as 2.6-fold for protoplasts obtained from the total leaf (L1 + L2 + L3) blades and total stalk (stalk lower + stalk upper) tissues (Figure 1C). These data indicate that the increase in ROS during the development from stalk lower to leaf blade can be attributed to the maturation of plastids and mitochondria (and probably to the ROS byproducts of electron transport chains used in both photosynthesis and respiration), rather than other parts of the cell where ROS can be produced.

Since DHR123 and CellROX report ROS in a general sense, we used other dyes to focus on specific types of ROS molecules. Hydrogen peroxide is a major non-radical oxygen species that is generated during photosynthetic and respiratory electron transport chain reactions, as well as in peroxisomes (Mhamdi et al., 2010). We used the Amplex red assay for H_2_O_2_ in organelles isolated from leaf and stalk tissues. The lowest H_2_O_2_ level was detected in stalk lower (plastids and mitochondria) and stalk (for protoplasts) tissues, with the highest level in L1 and leaf (Figures 1D–F and Supplementary Table S3).

Superoxide, a free-radical oxygen species, is also present in plant organelles. Using the mitochondrial-specific superoxide dye, MitoSOX red, we measured the superoxide level in mitochondria and protoplasts of leaf and stalk tissues (Figures 1G,H and Supplementary Table S5). We found that MitoSOX red, which was developed for mitochondria, did not work with isolated chloroplasts. The level of superoxide in mitochondria was higher by 2.3-fold for L1 than in stalk lower and 3-fold for leaf tissue than in stalk, respectively (Figures 1G,H). To summarize, both H_2_O_2_ and O_2_^•–^ were lower in stalk than leaves, so that the level of ROS clearly increases as the seedlings develop from stalk to leaf blade tissue.

### ROS Levels Are Higher in Light-Grown Than Dark-Grown Plants

We previously reported that orgDNA maintenance is influenced by responses to light signals: light that led to the greening of seedling leaves also triggered the demise of both ptDNA and mtDNA in maize (Oldenburg et al., 2006; Zheng et al., 2011; Kumar et al., 2014). Here, we test the hypothesis that the increased level of ROS in light-grown maize correlates with increased damage to orgDNA. We quantified ROS in plastids, mitochondria, and protoplasts from light- and dark-grown total leaf blade (L1 + L2 + L3) tissues, using the tissue with the lower amount of ROS as the baseline for comparison. The light/dark ratios were similar, ranging from 2.5 to 3.5, for general ROS (Figures 2A–C and Supplementary Table S2), H_2_O_2_ (Figures 2D–F and Supplementary Table S4), and O_2_^•–^ (Figures 2G,H and Supplementary Table S6). Although it might be expected that these light/dark ratios would be similar for plastids and protoplasts, it is notable that the light/dark ratios are also in the range of 2.5 to 3 for isolated, non-green mitochondria because there are no known photoreceptors in mitochondria.

### Change in Antioxidant Enzyme Activity During Development

Antioxidant enzymes can modulate the levels of ROS and reduce oxidative stress (Asada, 2006; Soares et al., 2019). The SOD enzymes remove superoxide by catalyzing its dismutation and reducing it to H_2_O_2_. Plants have MnSODs in the mitochondria and peroxisomes, Cu/ZnSODs in the chloroplast, peroxisomes, and cytosol, and FeSODs in the chloroplast (Alscher et al., 2002; Pilon et al., 2011). Our SOD assay measured all types of SOD activities. We measured SOD activity in organelles and protoplasts isolated from leaf and stalk tissues. As shown in Figures 3A–C and Supplementary Table S7, SOD activity was highest in organelles isolated from L1 and higher in protoplasts from leaf than from stalk.

The stability and accumulation of H_2_O_2_ are mainly influenced by the activity of the antioxidative system. In plants, several antioxidant enzymes metabolize H_2_O_2_. Ascorbate peroxidases (APX), peroxiredoxins, glutathione/thioredoxin peroxidases, glutathione *S*-transferases, and catalases are such enzymes (Cerny et al., 2018; Foyer, 2018). APXs have high specificity for H_2_O_2_, are present in chloroplast and mitochondria, and perform the final step of conversion of free radicals to water and oxygen (Maruta et al., 2016). Other peroxidases, such as peroxiredoxins, are localized to the cytosol, plastids, mitochondria, and peroxisomes in plants (Liebthal et al., 2018). Our peroxidase assay measured all types of peroxidase activities. We found more peroxidase activity in organelles isolated from stalk lower compared to organelles from L1 and more in protoplasts from stalk than from leaf (Figures 3D–F and Supplementary Table S9).

Catalase has high specificity for H_2_O_2_ and acts by the dismutation of two molecules of H_2_O_2_ to water and O_2_ (Mhamdi et al., 2010). Although the CAT-3 isoform of catalase was reported in maize mitochondria (Scandalios et al., 1980), and some catalase was reported in other cellular compartments including chloroplasts (Mhamdi et al., 2010), the peroxisome has been considered as the main location of catalase within plant cells, with mitochondrial/chloroplast catalase as possible contamination from broken peroxisomes (Corpas et al., 2017). Here, we report total cellular catalase activity in maize protoplasts prepared from stalk and leaf tissue. In our assays, catalase activity was higher in protoplasts from stalk than from leaf (Figure 3G and Supplementary Table S11).

In all antioxidant enzyme assays, the tissue with the lowest enzyme activity was used as the baseline for comparison with other tissues. Our assays suggest that high peroxidase and catalase activities result in the relatively low H_2_O_2_ level in the stalk, whereas low peroxidase and catalase activities result in high H_2_O_2_ levels in the leaf.

### Antioxidant Enzyme Activity in Light and Dark Conditions

The ROS level in the organelles isolated from leaf tissue of maize was higher for light-grown than dark-grown plants (Figure 2), and this difference may be attributed to lower antioxidant enzyme activity in the light. To test this idea, we measured the antioxidant activities of SOD, peroxidase, and catalase in leaves (L1 + L2 + L3) grown in light and dark conditions, as described above. We found 2–3.4 times higher SOD activity in the organelles and protoplasts of light-grown than dark-grown plants (Figures 4A–C and Supplementary Table S8). In contrast, the activity of peroxidase was 2–2.7 times lower for light-grown than dark-grown plants (Figures 4D–F and Supplementary Table S10). The catalase activity was 2.4 times lower in protoplasts from light compared to dark (Figure 4G and Supplementary Table S12).

### Levels of Small-Molecule Antioxidants

The non-enzymatic antioxidant system in plants includes low-mass metabolites like GSH, AsA, phenolic compounds, and proline. These antioxidants manage the ROS homeostasis by removing, transforming, or neutralizing the oxidant pool (Diaz-Vivancos et al., 2015; Smirnoff, 2018; Soares et al., 2019). To determine if small antioxidants are involved in maintaining low levels of ROS in maize, we quantified the levels of GSH and AsA in protoplasts isolated from leaf and stalk tissues. We found that the level of GSH was 2.2 times higher in stalk compared to leaf (Figure 3H and Supplementary Table S11). And we found that the level of AsA was 1.2 times higher in stalk than in leaf (Figure 3I and Supplementary Table S11).

We also performed the GSH and AsA assays in protoplasts isolated from the light-and dark-grown leaves. As shown in Figures 4H,I and Supplementary Table S12, both GSH and AsA levels were higher in the protoplasts isolated from the dark-grown plants. In protoplasts from the dark-grown leaves, the GSH level was 2.8 times higher, and the AsA level was 1.4 times higher than protoplasts from light-grown plants.

In summary (Table 2), we found higher levels of ROS (H_2_O_2_ and superoxide) and higher SOD activity in leaf than stalk tissues, but lower activities for peroxidase and catalase in leaf than stalk tissues. Light-grown leaves had higher levels of H_2_O_2_, superoxide, and SOD but lower activities of peroxidase and catalase than dark-grown leaves. Levels of GSH and AsA also changed, lower in light-grown leaf than in stalk tissue.

**TABLE 2:** Results summary for plastids, mitochondria, and protoplasts.

**Assay**	**Results**
General ROS	Leaf > stalk
	Light > dark
Hydrogen peroxide	Leaf > stalk
	Light > dark
Superoxide*	Leaf > stalk
	Light > dark
Superoxide dismutase	Leaf > stalk
	Light > dark
Peroxidase	Leaf < stalk
	Light < dark
Catalase**	Leaf < stalk
	Light < dark
Glutathione**	Leaf < stalk
	Light < dark
Ascorbic acid**	Leaf < stalk
	Light < dark

*Superoxide assay was not performed for plastids. **Assays were performed only for protoplasts.

### Levels of orgDNA Oxidative Damage Change During Development and in Light/Dark Conditions

Reactive oxygen species generate various modified DNA bases, and 7,8-dihydro-8-oxoguanine (8-oxoguanine, 8-oxoG) is the most common modified base found in DNA from bacteria, the eukaryotic nucleus, and mitochondria of animals, plants, and yeast (Imlay, 2013; Wallace, 2013; Bokhari and Sharma, 2019). During repair via the BER pathway, the damaged nucleoside, 8-hydroxydeoxyguanasine (8-OHdG), is released and has been used as a biomarker for oxidative stress (Shen et al., 2007). 8-oxoG lesions in DNA have also been assessed in rat liver cells by immunofluorescence (Kemeleva et al., 2006).

We used antibodies to 8-OHdG and a competitive ELISA method to measure the relative amounts of oxidative damage in orgDNA from maize tissues. In our ELISA assay, we found a higher level of 8-OHdG in ptDNA and mtDNA isolated from the blade of L1 compared to stalk lower tissue (Figures 5A,B and Supplementary Table S13). The level was 1.4–1.6 times higher in orgDNAs isolated from L1 compared to orgDNAs isolated from stalk lower. We also found 1.4 times higher 8-OHdG in the orgDNA of leaves from seedlings grown in the light than in the dark (Figures 5C,D and Supplementary Table S14). The higher 8-OHdG levels strongly suggest greater oxidative damage in the organelles from leaf than stalk.

**FIGURE 6:**
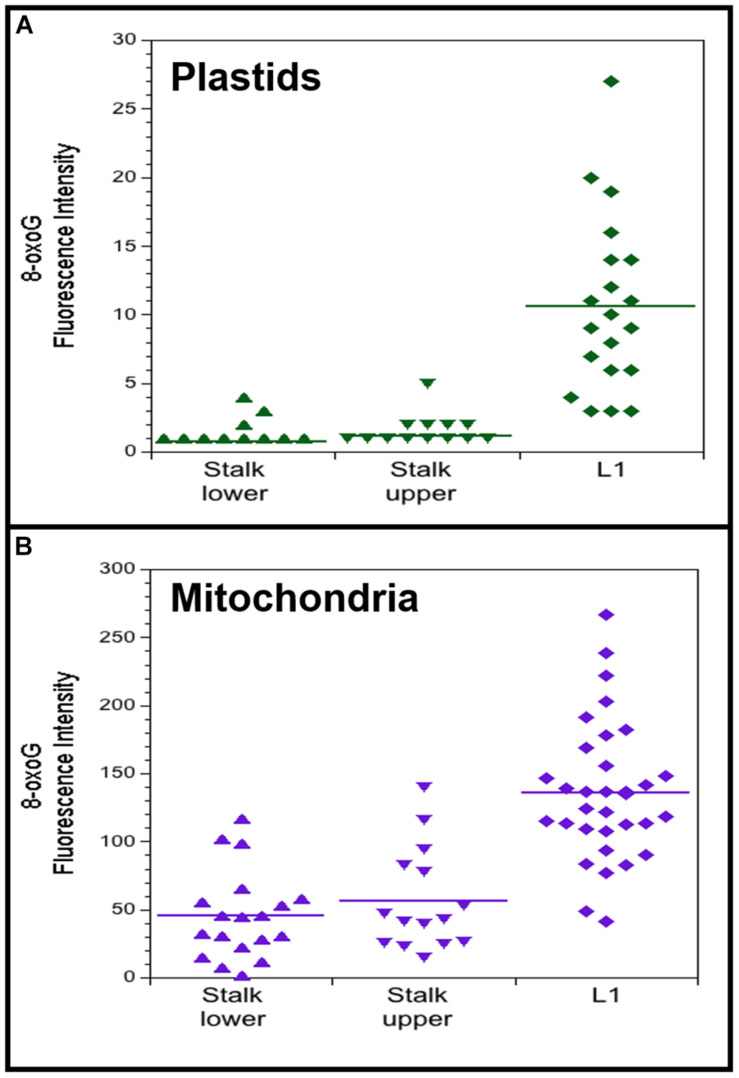
8-oxoguanine (8-oxoG) in maize plastids and mitochondria. Plastids **(A)** and mitochondria **(B)** were isolated from light-grown maize seedling tissues (Stalk lower, basal 1/3 of stalk; Stalk upper, upper 2/3 of stalk; L1, leaf 1). Fluorescence intensity was measured for individual plastids and mitochondria that were imaged by immunofluorescence microscopy using a primary antibody to 8-oxoG coupled with a secondary fluorescence antibody. All assays were performed at least three times. **(A)** The number of plastids measured and the average mean fluorescence intensity ± standard error was 22 and 0.8 ± 0.2 for Stalk lower, 18 and 1.2 ± 0.3 for Stalk upper, and 20 and 10.6 ± 1.4 for L1. **(B)** The number of mitochondria measured and the average mean fluorescence intensity ± standard error was 19 and 45 ± 7 for Stalk lower, 15 and 56 ± 10 for Stalk upper, and 33 and 136 ± 9 for L1. For both plastids and mitochondria, Stalk lower and Stalk upper are significantly different compared to L1 with *P*-value < 0.0001 using ANOVA with *post hoc* Tukey HSD, and there is no significant difference between Stalk lower and Stalk upper. Differences in the Fluorescence intensity may be attributed to different systems used to acquire images for plastids and mitochondria (see “Materials and Methods” section).

Immunofluorescence microscopy was performed using isolated plastids and mitochondria from light-grown maize tissues (stalk lower, stalk upper, and L1) with a primary antibody to 8-oxoG and a secondary antibody containing the fluorescent dye Alexa 488. For both plastids and mitochondria, the fluorescence intensity per organelle was higher for the leaf than the stalk tissues (Figures 6A,B). 8-OxoG immunofluorescence was also evaluated for plastids from light- and dark-grown entire seedling shoots (stalk and leaves). In this assay, the fluorescence intensity was assessed visually and scored as undetectable, weak, or high. For light, 75 out of 107 plastids were scored as “high”; for dark, 0 of 48 plastids were scored as “high.” Each of these results indicate that there are more 8-oxoG lesions in ptDNA from photosynthetically active leaf chloroplasts than non-photosynthetic stalk plastids.

## Discussion

Most of the changes we report for ROS and antioxidant agents during maize development might have been anticipated as a mitigating response to damage resulting from oxidative stress. In some cases, however, the anticipated damage-defense relationship was not observed suggesting a beneficial role for ROS unrelated to damage. We now consider how ROS and antioxidant agents may influence the maintenance or degradation of orgDNA during the transition from stem cell to leaf.

### ROS and Antioxidant Agents in Plant Cells and Organelles: An Overview

Although chloroplasts and mitochondria are the main sites of ROS production, ROS profoundly influence the chemistry in peroxisomes, cytosol, and vacuoles (Asada, 2006; Noctor and Foyer, 2016; Kohli et al., 2019). Cells also contain many protein and small-molecule antioxidant agents that both counteract oxidative stress and facilitate signaling the redox status of the cell to the nucleus, probably in the form of H_2_O_2_ (Cerny et al., 2018; Locato et al., 2018; Smirnoff and Arnaud, 2019; Soares et al., 2019). These include various SOD enzymes that convert the highly reactive O_2_^•–^ to the mobile but less reactive H_2_O_2_ (Alscher et al., 2002; Pilon et al., 2011), catalases that remove H_2_O_2_ (Zamocky et al., 2008; Corpas et al., 2017), and APXs and glutathione peroxidases (GTXs) (Maruta et al., 2016; Liebthal et al., 2018) that also remove H_2_O_2_, as well as GSH, AsA, and other small-molecule antioxidants (Noctor et al., 2012; Diaz-Vivancos et al., 2015; Smirnoff, 2018). The redox “objective” indicated by levels of these antioxidant agents, as well as oxygen content, seems to be directed at suppressing oxidative damage in the meristem while tolerating some oxidative damage in the green leaf. One consequence is seen as pristine orgDNA in the germline meristem and highly fragmented/damaged orgDNA in developing somatic cells.

### Relative and Absolute Levels of ROS and Antioxidant Agents

Our data are shown as the relative levels of several types of ROS and antioxidant agents, using the tissue with the lowest level as the reference point. Most assays report fluorescence units from reactive dyes or enzyme activity, neither of which provide concentration level. For example, we find that superoxide increases from stalk to green leaf (Figure 1G), but we do not know the molar concentration in either tissue. And superoxide levels were determined only for mitochondria, not for plastids, even in the protoplast assays. The assay for H_2_O_2_ does report concentration, although this is for a given volume of pooled organelles or protoplasts. For plastids from the stalk lower, the concentration ranged from 2.5–5.3 μM H_2_O_2_ and increased to 8.5–19.6 in the green leaf. Similar values were found for mitochondria, 3.3–4.3 in stalk lower and 8.2–11.7 in leaf. Not surprisingly, the cellular H_2_O_2_ concentration was higher, 18.7–24.5 μM for protoplasts from light-grown leaves. The molarity of H_2_O_2_ extracted from various plants has been reported, but these values enormously exceed the values for animal cells, probably do the extremely high levels in the apoplastic parts of plant tissues (cell walls and intercellular spaces) (Foyer and Noctor, 2016; Noctor et al., 2018). Previous estimates of the absolute concentrations of ROS molecules within plant cells are problematic, in part due to technical difficulties, and even newer methods employing genetically engineered ROS-sensor proteins (HyPer) only report relative differences in ROS levels between samples (such as control and high light exposure) or along a cellular gradient (Exposito-Rodriguez et al., 2017; Smirnoff and Arnaud, 2019). Our data were obtained with isolated organelles and protoplasts, including many wash steps, so that apoplastic sources do not affect our ROS and antioxidant data. Maize is a C4 plant containing both mesophyll and bundle sheath cells. The isolated organelles and protoplasts prepared using our methods are mostly derived from mesophyll cells (Kumar et al., 2015), so that our ROS and antioxidant data represent a specific subset of differentiated maize cells. Whereas accurate measurements of the concentrations of ROS molecules and antioxidants in plant cell compartments are needed to better understand the influence of ROS in oxidative stress and signaling, comparisons of the relative levels in isolated organelles can provide insights, as described below.

### Isolated Organelles and Organelles in the Plant

**FIGURE 7:**
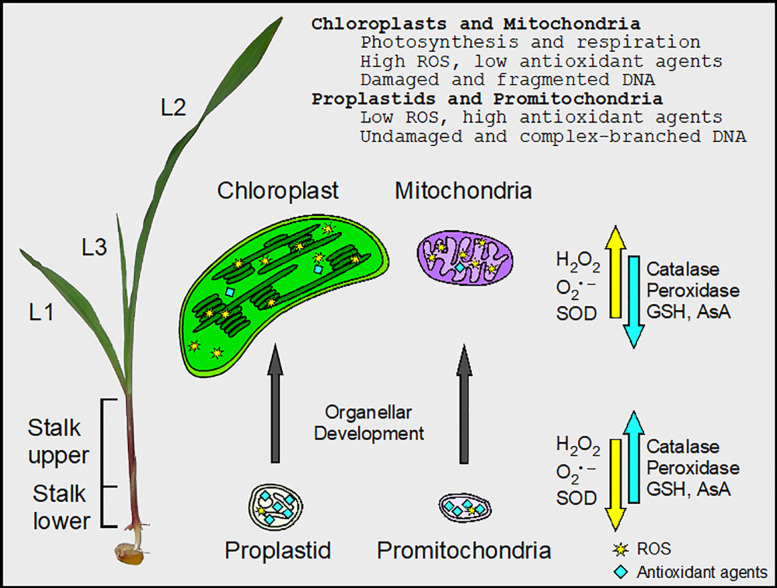
Changes in ROS and antioxidant agents during maize chloroplast and mitochondrial development. There is a gradient in cellular and organellar development from the basal meristem (Stalk lower) to the fully expanded leaf blade in maize. In the undeveloped proplastids and promitochondria, the activity of peroxidase was high, facilitating the maintenance of minimal ROS levels and protecting orgDNA from oxidative damage. In chloroplasts and mitochondria, there were high levels of ROS, the byproducts of photosynthesis and respiration. Superoxide dismutase (SOD) converted some superoxide (O_2_^•–^) to H_2_O_2_, but the activity of peroxidase was low. Thus, the orgDNA was subjected to extensive oxidative damage and became fragmented. Levels of glutathione (GSH), ascorbic acid (AsA), and catalase activity were obtained from protoplasts from the entire stalk and leaf blades (L1–L3), whereas both protoplasts and isolated organelles were used to assay ROS and enzyme activities for SOD and peroxidase.

Any biochemical property may be altered by removing molecules or organelles from intact tissue before analysis, and the nature of ROS can make measurements especially challenging (Noctor et al., 2016). In order to mitigate effects of inadvertent oxidation on subsequent assays of ROS, antioxidant enzymes, and orgDNA damage, we employed low temperature and reducing conditions during the isolation of organelles. Furthermore, our data are reported as relative values among tissues handled and analyzed in parallel, so that potential isolation artifacts were controlled. The overall conclusions, summarized in Figure 7, present a relationship between redox status and DNA damage that would not be anticipated from artifactual data.

### The Transition From Stem Cells to Leaf Cells

During maize leaf development, new cells arise from the basal meristem, begin expansion/elongation in the stalk region, and become fully differentiated in the leaf blade. The developing cells in the stalk are in an etiolated state, shielded from light first by the coleoptile and later by the outer leaf sheath. Only after the leaf blade tip emerges are the cells exposed to light and begin the final differentiation process to photosynthetic capability. It is this final step where a sudden increase in ROS due to photosynthesis may result in oxidative stress or may merely be part of the normal cellular signaling process.

Antioxidant agents are usually considered as defensive agents that relieve oxidative stress and damage caused by high levels of ROS. We find that superoxide, SOD, and 8-oxoG all increase during leaf development (Table 2 and Figure 7), a result consistent with a damage-response function for the antioxidant enzyme SOD. But perhaps SOD does not act only to reduce oxidative stress when it converts superoxide to H_2_O_2_ for two reasons. Since H_2_O_2_ is more stable than superoxide, a consequence of SOD activity is the replacement of one type of ROS with a more persistent type. The neutralization of superoxide as a damaging agent involves a second step in which H_2_O_2_ is removed by catalase and peroxidases. But as the leaf develops from the lower stalk, the increase in cellular H_2_O_2_ is accompanied by a *decrease* in cellular catalase and peroxidase activities and GSH and AsA levels. The second reason is that mutations that either over-express or reduce levels of some SODs reveal that these proteins have only minor roles in photoprotection or protection from oxidative damage (Alscher et al., 2002; Pilon et al., 2011). However, severe developmental defects were observed in mutants deficient for two plastid FeSODs, and H_2_O_2_ was proposed to coordinate chloroplast-nuclear gene expression (Myouga et al., 2008). Therefore, a major function of SOD may be to convert an immobile ROS species (superoxide) to a mobile species (H_2_O_2_), which is the likely signaling molecule that communicates the redox status of the cell to the nucleus (Brunkard and Burch-Smith, 2018; Mullineaux et al., 2018; Janku et al., 2019). In the lower stalk, however, the low level of SOD combined with the high activities of catalase and peroxidase and higher levels of GSH and AsA (Figure 3) serves to maintain a relatively high ratio of superoxide to H_2_O_2_ that is thought to be required for “stemness” in the stem cells of the shoot apical meristem of Arabidopsis (Zeng et al., 2017; Yang et al., 2018).

To summarize, our data support a signaling role for ROS in the lower stalk to maintain the stem cells (that later lead to the gametes) and in leaf cells developing photosynthetic capacity. Furthermore, mitochondria and plastids may comprise the major source of the H_2_O_2_ signaling molecules produced during leaf development (Figures 1, 7).

### Damage and Repair of Organellar DNA

In maize, the DNA molecules in mitochondria and plastids isolated from the stalk lower tissue are pristine, as expected for stem cells, but these orgDNAs are highly degraded in green leaves (Oldenburg and Bendich, 2004, 2015; Oldenburg et al., 2013). Our present data show that during leaf development there is an increase in ROS and 8-oxoG (representing orgDNA damage). We infer that the demise of orgDNA begins with increased ROS production and that damaged-but-not-repaired orgDNA is degraded by default as occurs in *Escherichia coli* (Skarstad and Boye, 1993). Our data show that ROS, H_2_O_2_, superoxide, and SOD levels are lower in dark-grown leaves than in those grown in the light. Previously, we reported that the amount and molecular integrity of ptDNA rapidly decline upon transfer of maize seedlings from dark to light growth conditions (Zheng et al., 2011). We speculate that in the dark, the ROS byproducts of respiration lead to mtDNA damage and that a small amount of H_2_O_2_ signals the nucleus to express and deliver DNA repair proteins to the mitochondria and plastids. In the light, the green cells of the maize leaf blade produce sufficient ATP by photophosphorylation and no longer require transcription from either mtDNA to support respiration or ptDNA to support photosynthesis. A larger amount of H_2_O_2_ from the chloroplasts now signals the nucleus to cut off the supply of DNA repair proteins (such as RecA) and the damaged DNA in both organelles disintegrates. [The theoretical problem of continued photosynthesis in single-season grasses, such as maize, using long-lived mRNAs without the support of functional ptDNA has been considered elsewhere (Oldenburg and Bendich, 2015)].

The changes in ROS and antioxidant enzyme levels (SOD and peroxidase) during maize seedling development and under light and dark growth conditions show the same trends in both plastids and mitochondria. However, since mitochondria have no known light receptors, it is unclear why the mtDNA suffers the same light-induced demise as does ptDNA. One possible explanation comes from cultured human retinal pigment epithelial cells: the electron transport chain generates ROS when cells are exposed to blue light (King et al., 2004). In maize, we reported lower amounts of DNA per plastid in blue light than in white light and concluded that blue light suppresses ptDNA replication/repair and induces degradation (Oldenburg et al., 2006). One way for the nucleus to coordinate organellar activities is expression of dual-targeted proteins (those delivered to both plastids and mitochondria), including the replication/repair protein RecA (Oldenburg and Bendich, 2015). Thus, regardless of the organellar source of H_2_O_2_, considered the main signaling molecule, the nucleus perceives the redox status of the cell and dictates the fate of the orgDNA.

Although mechanisms for orgDNA repair in plants have been addressed recently (Baruch-Torres and Brieba, 2017; Garcia-Medel et al., 2019), additional insight may be found elsewhere. The “SOS response” in *E. coli* is initiated by accumulation of single-stranded DNA (ssDNA) during replication of DNA containing lesions (Maslowska et al., 2019). For both bacteria and eukaryotes, Bantele and Pfander (2019) propose a mechanism for responding to DNA damage that is based on the persistence of ssDNA: (1) repair locally if the damage level is low and ssDNA is short-lived; and (2) halt cell division until global repair of high-level damage is accomplished. The overall amount of ssDNA in a given cell must exceed a threshold to activate a DNA damage “checkpoint” for cell division. How might these precedents influence the integrity of orgDNA in plants?

Pristine DNA in the gametes is required for maintaining the lineage of a sexually reproducing organism. High-quality DNA is maintained by damage repair in a eukaryote with a single cell type, such as yeast or *Chlamydomonas*, regardless of the metabolic cost of DNA repair. But for a species with embryonic development (like maize), the high cost of DNA repair can be reduced by powering germline cells with “quiet” metabolism (neither respiration nor photosynthesis) and the somatic cells with “active” metabolism (both respiration and photosynthesis) (Bendich, 2010). Of course, full repair of DNA damage is required in the meristem. But low oxygen, low H_2_O_2_, and high peroxidase and catalase reduce the potential for oxidative DNA damage, lowering the cost to repair both orgDNA and nuclear DNA. Thus, the local repair of a low level of damage to orgDNA would suffice, and repair pathways including BER, HR, and perhaps MMEJ operate in both plastids and mitochondria in some plants (Boesch et al., 2011; Garcia-Medel et al., 2019). Maize was not among those investigated, although its plastid proteome does contain DNA repair proteins, and most of these (including RecA) were found in greater abundance in the proplastids at the leaf base than in chloroplasts at the leaf tip (Majeran et al., 2012). Although wild type Arabidopsis ptDNA contains some ssDNA, a large increase in ssDNA regions was found in a *cprecA* mutant (Rowan et al., 2010). In maize leaf, if RecA expression is turned off following ROS signaling, ssDNA should accumulate in orgDNA leading to its demise. For somatic cells in the leaf, the plant can “afford” to not repair all of the high oxidatively damaged copies of their organellar genomes, thereby reducing the high cost of orgDNA repair, although repair of nuclear DNA is required for cellular homeostasis and checkpoint control of cell division.

## Conclusion

Studies on ROS in plants typically consider the effects following biotic and abiotic stress. Here, we focus on changes in ROS and antioxidant agents under normal, non-stressful growth conditions and show that the ROS levels increase in whole cells and in plastids and mitochondria during maize leaf development. Although we report changes in the relative levels of ROS and antioxidant agents, a deeper understanding of ROS in oxidative stress and signaling may be gained when new methods are developed to measure absolute concentrations. Previously, we showed differences in the maintenance and degradation of ptDNA between maize and other plants, including tobacco and Arabidopsis (Shaver et al., 2006; Rowan and Bendich, 2009). These differences could be due to variations in response to ROS signaling. We propose that orgDNA degradation in maize leaf is a result of an increase in oxidative damage to orgDNA and ROS-signaling that leads to a decrease in orgDNA repair systems.

## Data Availability Statement

All datasets generated for this study are included in the article/Supplementary Material.

## Author Contributions

DT and AN performed the experiments. DT, AN, and DO analyzed the data. DT, DO, and AB wrote the manuscript. All authors have read and approved the final version of the manuscript.

## Conflict of Interest

The authors declare that the research was conducted in the absence of any commercial or financial relationships that could be construed as a potential conflict of interest.
