# Supplementary material for: Reactive Oxygen Species, Antioxidant Agents, and DNA Damage in Developing Maize Mitochondria and Plastids
Source: Front Plant Sci. 2020 May 19;11:596. doi: 10.3389/fpls.2020.00596 (PMC7248337; doi:10.3389/fpls.2020.00596)
Supplement: Supplementary file 1 [file Data_Sheet_1.DOCX]

ROS – Supplemental Material

**Quantification of ROS and antioxidant agents in maize plastids, mitochondria, and protoplasts**

Plastids, mitochondria and protoplasts were isolated from maize seedling tissues as described in Methods and equal volumes of plastids, mitochondria or protoplasts were used for each set of assays. Three biological replicates (Assay 1-3) using pooled chloroplasts, mitochondria or protoplasts were done for each assay and with three technical replicates for each biological replicate. In the tables given below, assay values were measured as fluorescence units (FU), concentration (uM; ng/mL), enzyme activity (U/mL; mU/mL) or small antioxidants levels (nM or µM), and Mean ± standard error (SE) for Assays 1-3 is given in the tables. These values were then converted to give the level relative to the tissue with the lowest value which is set at one (Methods). The Mean Relative level ± SE (calculated as given below) in each assay shows the average of the three biological replicates. Unless specified in the table, the Mean Relative level of different tissues (such as comparing Stalk lower to Stalk upper, Stalk lower to L1, etc.) in each table are significantly different from each other at P value ≤0.05 as analyzed by the ANOVA statistic test with post-hoc analysis using Tukey’s HSD. Data with P value > 0.05 are indicated as not significant at P value ≤0.05 by asterisks and footnotes in their respective tables.

The standard error of the ratio (i.e. the Mean Relative level) was determined using the following equation:

$$\boldsymbol{SE}\left( \frac{\boldsymbol{x}}{\boldsymbol{y}} \right)\boldsymbol{=}\left( \frac{\boldsymbol{x}}{\boldsymbol{y}} \right)\sqrt{\left( \frac{\boldsymbol{SE}\left( \boldsymbol{x} \right)}{\boldsymbol{x}} \right)^{\boldsymbol{2}}}\boldsymbol{+}\left( \frac{\boldsymbol{SE}\left( \boldsymbol{y} \right)}{\boldsymbol{y}} \right)^{\boldsymbol{2}}$$

where x, SE(x), y, and SE(y) are the Mean value (such as FU as given in the examples below) and standard error from Assays 1-3.

The following examples are from Table S1:

For Stalk lower: x = 1152; SE(x) = 88; y = 1152; SE(y) = 88

$$\boldsymbol{SE}\left( \frac{\boldsymbol{1152}}{\boldsymbol{1152}} \right)\boldsymbol{=}\left( \frac{\boldsymbol{1152}}{\boldsymbol{1152}} \right)\sqrt{\left( \frac{\boldsymbol{SE}\left( \boldsymbol{88} \right)}{\boldsymbol{1152}} \right)^{\boldsymbol{2}}}\boldsymbol{+}\left( \frac{\boldsymbol{SE}\left( \boldsymbol{88} \right)}{\boldsymbol{1152}} \right)^{\boldsymbol{2}}\boldsymbol{=0.11}$$

For Stalk upper: x = 1938; SE(x) = 195; y = 1152; SE(y) = 88

$$\boldsymbol{SE}\left( \frac{\boldsymbol{1938}}{\boldsymbol{1152}} \right)\boldsymbol{=}\left( \frac{\boldsymbol{1938}}{\boldsymbol{1152}} \right)\sqrt{\left( \frac{\boldsymbol{SE}\left( \boldsymbol{195} \right)}{\boldsymbol{1938}} \right)^{\boldsymbol{2}}}\boldsymbol{+}\left( \frac{\boldsymbol{SE}\left( \boldsymbol{88} \right)}{\boldsymbol{1152}} \right)^{\boldsymbol{2}}\boldsymbol{=0.21}$$

For L1: x = 3010; SE(x) = 241; y = 1152; SE(y) = 88

$$\boldsymbol{SE}\left( \frac{\boldsymbol{3010}}{\boldsymbol{1152}} \right)\boldsymbol{=}\left( \frac{\boldsymbol{3010}}{\boldsymbol{1152}} \right)\sqrt{\left( \frac{\boldsymbol{SE}\left( \boldsymbol{241} \right)}{\boldsymbol{3010}} \right)^{\boldsymbol{2}}}\boldsymbol{+}\left( \frac{\boldsymbol{SE}\left( \boldsymbol{88} \right)}{\boldsymbol{1152}} \right)^{\boldsymbol{2}}\boldsymbol{=0.29}$$

**Table S1 ROS levels during maize development**

| **Tissue** | **Plastids** | | | | | | | |
| --- | --- | --- | --- | --- | --- | --- | --- | --- |
|  | **Assay 1** | | **Assay 2** | | **Assay 3** | | **Mean**  **FU**  **± SE** | **Mean**  **Relative level**  **± SE** |
|  | FU | Relative ROS level | FU | Relative ROS level | FU | Relative ROS level |  |  |
| **Stalk lower** | 1305 | 1.0 | 999 | 1.0 | 1151 | 1.0 | 1152  ± 88 | 1.0  ± 0.11 |
| **Stalk upper** | 2303 | 1.8 | 1635 | 1.6 | 1876 | 1.6 | 1938  ± 195 | 1.7  ±0.21 |
| **L1** | 3399 | 2.6 | 2569 | 2.6 | 3061 | 2.7 | 3010  ± 241 | 2.6  ± 0.29 |

| **Tissue** | **Mitochondria** | | | | | | | |
| --- | --- | --- | --- | --- | --- | --- | --- | --- |
|  | **Assay 1** | | **Assay 2** | | **Assay 3** | | **Mean**  **FU**  **± SE** | **Mean**  **Relative level**  **± SE** |
|  | FU | Relative ROS level | FU | Relative ROS level | FU | Relative ROS level |  |  |
| **Stalk lower** | 262 | 1.0 | 435 | 1.0 | 364 | 1.0 | 354  ±50 | 1.0^*^  ±0.20 |
| **Stalk upper** | 366 | 1.4 | 721 | 1.7 | 451 | 1.2 | 513  ±107 | 1.4^*^  ±0.37 |
| **L1** | 717 | 2.7 | 1138 | 2.6 | 1039 | 2.9 | 965  ±127 | 2.7  ±0.53 |

| **Tissue** | **Protoplasts** | | | | | | | |
| --- | --- | --- | --- | --- | --- | --- | --- | --- |
|  | **Assay 1** | | **Assay 2** | | **Assay 3** | | **Mean**  **FU**  **± SE** | **Mean**  **Relative level**  **± SE** |
|  | FU | Relative ROS level | FU | Relative ROS level | FU | Relative ROS level |  |  |
| **Stalk** | 278 | 1.0 | 340 | 1.0 | 250 | 1.0 | 332  ±29 | 1.0  ±0.12 |
| **Leaf** | 709 | 2.5 | 815 | 2.4 | 722 | 2.9 | 869  ±112 | 2.6  ±0.41 |

**Table S2 ROS levels under light and dark growth conditions**

| **Tissue** | **Plastids** | | | | | | | |
| --- | --- | --- | --- | --- | --- | --- | --- | --- |
|  | **Assay 1** | | **Assay 2** | | **Assay 3** | | **Mean**  **FU**  **± SE** | **Mean**  **Relative level**  **± SE** |
|  | FU | Relative ROS level | FU | Relative ROS level | FU | Relative ROS level |  |  |
| **Dark** | 1532 | 1.0 | 1446 | 1.0 | 1524 | 1.0 | 1500  ±27 | 1.0  ±0.03 |
| **Light** | 3864 | 2.5 | 3692 | 2.6 | 3903 | 2.6 | 3820  ±65 | 2.5  ±0.06 |

| **Tissue** | **Mitochondria** | | | | | | | |
| --- | --- | --- | --- | --- | --- | --- | --- | --- |
|  | **Assay 1** | | **Assay 2** | | **Assay 3** | | **Mean**  **FU**  **± SE** | **Mean**  **Relative level**  **± SE** |
|  | FU | Relative ROS level | FU | Relative ROS level | FU | Relative ROS level |  |  |
| **Dark** | 470 | 1.0 | 537 | 1.0 | 275 | 1.0 | 427  ±78 | 1.0  ±0.3 |
| **Light** | 1434 | 3.1 | 1223 | 2.3 | 1084 | 3.8 | 1247  ±102 | 3.0  ±0.6 |

| **Tissue** | **Protoplasts** | | | | | | | |
| --- | --- | --- | --- | --- | --- | --- | --- | --- |
|  | **Assay 1** | | **Assay 2** | | **Assay 3** | | **Mean**  **FU**  **± SE** | **Mean**  **Relative level**  **± SE** |
|  | FU | Relative ROS level | FU | Relative ROS level | FU | Relative ROS level |  |  |
| **Dark** | 343 | 1.0 | 398 | 1.0 | 517 | 1.0 | 1228  ±97 | 1.0  ±0.2 |
| **Light** | 1046 | 3.1 | 1260 | 3.2 | 1377 | 2.7 | 419  ±51 | 3.0  0.4 |

**Table S3 H_2_O_2_ levels during maize development**

| **Tissue** | **Plastids** | | | | | | | |
| --- | --- | --- | --- | --- | --- | --- | --- | --- |
|  | **Assay 1** | | **Assay 2** | | **Assay 3** | | **Mean**  **µM**  **± SE** | **Mean**  **Relative level**  **± SE** |
|  | H_2_O_2_  (µM) | Relative H_2_O_2_ level | H_2_O_2_  (µM) | Relative H_2_O_2_ level | H_2_O_2_  (µM) | Relative H_2_O_2_ level |  |  |
| **Stalk lower** | 5.3 | 1.0 | 4.0 | 1.0 | 2.3 | 1.0 | 3.9  ±0.9 | 1.0  ±0.3 |
| **Stalk upper** | 11.9 | 2.3 | 9.7 | 2.4 | 5.3 | 2.3 | 9.0  ±1.9 | 2.3  ±0.7 |
| **L1** | 19.6 | 3.7 | 14.2 | 3.5 | 8.5 | 3.8 | 14.1  ±3.2 | 3.7  ±1.2 |

| **Tissue** | **Mitochondria** | | | | | | | |
| --- | --- | --- | --- | --- | --- | --- | --- | --- |
|  | **Assay 1** | | **Assay 2** | | **Assay 3** | | **Mean**  **µM**  **± SE** | **Mean**  **Relative level**  **± SE** |
|  | H_2_O_2_  (µM) | Relative H_2_O_2_ level | H_2_O_2_  (µM) | Relative H_2_O_2_ level | H_2_O_2_  (µM) | Relative H_2_O_2_ level |  |  |
| **Stalk lower** | 3.7 | 1.0 | 3.3 | 1.0 | 4.0 | 1.0 | 10.2  ±0.2 | 1.0  ±0.1 |
| **Stalk upper** | 5.6 | 1.5 | 6.2 | 1.7 | 5.8 | 1.6 | 5.8  ±0.2 | 1.6  ±0.1 |
| **L1** | 10.6 | 2.9 | 8.2 | 2.5 | 11.7 | 3.0 | 3.7  ±1.0 | 2.8  ±0.3 |

| **Tissue** | **Protoplasts** | | | | | | | |
| --- | --- | --- | --- | --- | --- | --- | --- | --- |
|  | **Assay 1** | | **Assay 2** | | **Assay 3** | | **Mean**  **µM**  **± SE** | **Mean**  **Relative level**  **± SE** |
|  | H_2_O_2_  (µM) | Relative H_2_O_2_ level | H_2_O_2_  (µM) | Relative H_2_O_2_ level | H_2_O_2_  (µM) | Relative H_2_O_2_ level |  |  |
| **Stalk** | 9.3 | 1.0 | 8.1 | 1.0 | 9.3 | 1.0 | 8.9  ±0.4 | 1.0  ±0.1 |
| **Leaf** | 22.4 | 2.4 | 18.7 | 2.3 | 24.5 | 2.6 | 21.9  ±1.7 | 2.4  0.2 |

**Table S4 H_2_O_2_ levels under light and dark growth conditions**

| **Tissue** | **Plastids** | | | | | | | |
| --- | --- | --- | --- | --- | --- | --- | --- | --- |
|  | **Assay 1** | | **Assay 2** | | **Assay 3** | | **Mean**  **µM**  **± SE** | **Mean**  **Relative level**  **± SE** |
|  | H_2_O_2_  (µM) | Relative H_2_O_2_ level | H_2_O_2_  (µM) | Relative H_2_O_2_ level | H_2_O_2_  (µM) | Relative H_2_O_2_ level |  |  |
| **Dark** | 14 | 1.0 | 11 | 1.0 | 10 | 1.0 | 12  ±1 | 1.0  ±0.1 |
| **Light** | 45 | 3.1 | 38 | 3.2 | 31 | 3.3 | 38  ±4 | 3.2  ±0.5 |

| **Tissue** | **Mitochondria** | | | | | | | |
| --- | --- | --- | --- | --- | --- | --- | --- | --- |
|  | **Assay 1** | | **Assay 2** | | **Assay 3** | | **Mean**  **µM**  **± SE** | **Mean**  **Relative level**  **± SE** |
|  | H_2_O_2_  (µM) | Relative H_2_O_2_ level | H_2_O_2_  (µM) | Relative H_2_O_2_ level | H_2_O_2_  (µM) | Relative H_2_O_2_ level |  |  |
| **Dark** | 7 | 1.0 | 17 | 1.0 | 22 | 1.0 | 15  ±4 | 1.0  ±0.4 |
| **Light** | 23 | 3.4 | 59 | 3.5 | 45 | 2.1 | 42  ±10 | 3.0  ±1.0 |

| **Tissue** | **Protoplasts** | | | | | | | |
| --- | --- | --- | --- | --- | --- | --- | --- | --- |
|  | **Assay 1** | | **Assay 2** | | **Assay 3** | | **Mean**  **µM**  **± SE** | **Mean**  **Relative level**  **± SE** |
|  | H_2_O_2_  (µM) | Relative H_2_O_2_ level | H_2_O_2_  (µM) | Relative H_2_O_2_ level | H_2_O_2_  (µM) | Relative H_2_O_2_ level |  |  |
| **Dark** | 18 | 1.0 | 17 | 1.0 | 19 | 1.0 | 18  ±0.4 | 1.0  ±0.03 |
| **Light** | 56 | 3.1 | 63 | 3.7 | 71 | 3.8 | 63  ±4.0 | 3.5  ±0.24 |

**Table S5 Superoxide levels during maize development**

| **Tissue** | **Mitochondria** | | | | | | | |
| --- | --- | --- | --- | --- | --- | --- | --- | --- |
|  | **Assay 1** | | **Assay 2** | | **Assay 3** | | **Mean**  **FU**  **± SE** | **Mean**  **Relative level**  **± SE** |
|  | FU | Relative superoxide level | FU | Relative superoxide level | FU | Relative superoxide level |  |  |
| **Stalk lower** | 1174 | 1.0 | 2088 | 1.0 | 1618 | 1.0 | 1627  ±264 | 1.0^*^  ±0.23 |
| **Stalk upper** | 1423 | 1.2 | 2296 | 1.1 | 1808 | 1.1 | 1842  ±253 | 1.1^*^  ±0.24 |
| **L1** | 2895 | 2.5 | 5039 | 2.4 | 3316 | 2.0 | 3750  ±656 | 2.3  ±0.55 |

**** Not significant at P value <0.05***

| **Tissue** | **Protoplasts** | | | | | | | |
| --- | --- | --- | --- | --- | --- | --- | --- | --- |
|  | **Assay 1** | | **Assay 2** | | **Assay 3** | | **Mean**  **FU**  **± SE** | **Mean**  **Relative level**  **± SE** |
|  | FU | Relative superoxide level | FU | Relative superoxide level | FU | Relative superoxide level |  |  |
| **Stalk** | 1871 | 1.0 | 2319 | 1.0 | 1324 | 1.0 | 1838  ±289 | 1.0  ±0.2 |
| **Leaf** | 5199 | 2.8 | 7771 | 3.4 | 3898 | 2.9 | 5623  ±1138 | 3.0  ±0.8 |

**Table S6 Superoxide levels under light and dark growth conditions**

| **Tissue** | **Mitochondria** | | | | | | | |
| --- | --- | --- | --- | --- | --- | --- | --- | --- |
|  | **Assay 1** | | **Assay 2** | | **Assay 3** | | **Mean**  **FU**  **± SE** | **Mean**  **Relative level**  **± SE** |
|  | FU | Relative superoxide level | FU | Relative superoxide level | FU | Relative superoxide level |  |  |
| **Dark** | 1278 | 1.0 | 1264 | 1.0 | 1375 | 1.0 | 1306  ±35 | 1.0  ±0.04 |
| **Light** | 3408 | 2.7 | 3340 | 2.6 | 2998 | 2.2 | 3249  ±127 | 2.5  ±0.12 |

| **Tissue** | **Protoplasts** | | | | | | | |
| --- | --- | --- | --- | --- | --- | --- | --- | --- |
|  | **Assay 1** | | **Assay 2** | | **Assay 3** | | **Mean**  **FU**  **± SE** | **Mean**  **Relative level**  **± SE** |
|  | FU | Relative superoxide level | FU | Relative superoxide level | FU | Relative superoxide level |  |  |
| **Dark** | 1308 | 1.0 | 1850 | 1.0 | 1394 | 1.0 | 1527  ±168 | 1.0  ±0.16 |
| **Light** | 3825 | 2.9 | 4353 | 2.8 | 3900 | 2.4 | 4026  ±165 | 2.7  ±0.31 |

**Table S7 SOD activity during maize development**

| **Tissue** | **Plastids** | | | | | | | |
| --- | --- | --- | --- | --- | --- | --- | --- | --- |
|  | **Assay 1** | | **Assay 2** | | **Assay 3** | | **Mean**  **U/mL**  **± SE** | **Mean**  **Relative level**  **± SE** |
|  | U/mL | Relative SOD activity | U/mL | Relative SOD activity | U/mL | Relative SOD activity |  |  |
| **Stalk lower** | 0.16 | 1.0 | 0.19 | 1.0 | 0.23 | 1.0 | 0.19  ±0.02 | 1.0  ±0.15 |
| **Stalk upper** | 0.26 | 1.6 | 0.37 | 1.9 | 0.45 | 2.0 | 0.36  ±0.05 | 1.9  ±0.32 |
| **L1** | 0.54 | 3.4 | 0.63 | 3.3 | 0.66 | 2.9 | 0.61  ±0.04 | 3.2  ±0.39 |

| **Tissue** | **Mitochondria** | | | | | | | |
| --- | --- | --- | --- | --- | --- | --- | --- | --- |
|  | **Assay 1** | | **Assay 2** | | **Assay 3** | | **Mean**  **U/mL**  **± SE** | **Mean**  **Relative level**  **± SE** |
|  | U/mL | Relative SOD activity | U/mL | Relative SOD activity | U/mL | Relative SOD activity |  |  |
| **Stalk lower** | 0.07 | 1.0 | 0.11 | 1.0 | 0.12 | 1.0 | 0.10  ±0.01 | 1.0***  ± 0.15 |
| **Stalk upper** | 0.09 | 1.3 | 0.11 | 1.0 | 0.14 | 1.2 | 0.11  ±0.01 | 1.2***  ± 0.16 |
| **L1** | 0.14 | 2.0 | 0.18 | 1.7 | 0.17 | 1.4 | 0.16  ± 0.01 | 1.7  ±0.20 |

**** Not significant at P value <0.05***

| **Tissue** | **Protoplasts** | | | | | | | |
| --- | --- | --- | --- | --- | --- | --- | --- | --- |
|  | **Assay 1** | | **Assay 2** | | **Assay 3** | | **Mean**  **U/mL**  **± SE** | **Mean**  **Relative level**  **± SE** |
|  | U/mL | Relative SOD activity | U/mL | Relative SOD activity | U/mL | Relative SOD activity |  |  |
| **Stalk** | 0.06 | 1.0 | 0.09 | 1.0 | 0.06 | 1.0 | 0.07  ±0.01 | 1.0  ±0.20 |
| **Leaf** | 0.12 | 2.0 | 0.14 | 1.6 | 0.16 | 2.6 | 0.14  ±0.01 | 2.0  ±0.31 |

**Table S8 SOD activity during under light and dark growth conditions**

| **Tissue** | **Plastids** | | | | | | | |
| --- | --- | --- | --- | --- | --- | --- | --- | --- |
|  | **Assay 1** | | **Assay 2** | | **Assay 3** | | **Mean**  **U/mL**  **± SE** | **Mean**  **Relative level**  **± SE** |
|  | U/mL | Relative SOD activity | U/mL | Relative SOD activity | U/mL | Relative SOD activity |  |  |
| **Dark** | 1.54 | 1.0 | 2.15 | 1.0 | 2.03 | 1.0 | 1.91  ±0.18 | 1.0  ±0.13 |
| **Light** | 4.90 | 3.2 | 7.75 | 3.6 | 6.87 | 3.4 | 6.50  ±0.84 | 3.4  ±0.55 |

| **Tissue** | **Mitochondria** | | | | | | | |
| --- | --- | --- | --- | --- | --- | --- | --- | --- |
|  | **Assay 1** | | **Assay 2** | | **Assay 3** | | **Mean**  **U/mL**  **± SE** | **Mean**  **Relative level**  **± SE** |
|  | U/mL | Relative SOD activity | U/mL | Relative SOD activity | U/mL | Relative SOD activity |  |  |
| **Dark** | 0.75 | 1.0 | 1.19 | 1.0 | 1.31 | 1.0 | 1.08  ±0.17 | 1.0  ±0.22 |
| **Light** | 1.89 | 2.5 | 1.93 | 1.6 | 2.66 | 2.0 | 2.16  ±0.25 | 2.0  ±0.39 |

| **Tissue** | **Protoplasts** | | | | | | | |
| --- | --- | --- | --- | --- | --- | --- | --- | --- |
|  | **Assay 1** | | **Assay 2** | | **Assay 3** | | **Mean**  **U/mL**  **± SE** | **Mean**  **Relative level**  **± SE** |
|  | U/mL | Relative SOD activity | U/mL | Relative SOD activity | U/mL | Relative SOD activity |  |  |
| **Dark** | 1.19 | 1.0 | 1.75 | 1.0 | 2.06 | 1.0 | 1.67  ±0.25 | 1.0  ±0.21 |
| **Light** | 2.73 | 2.3 | 3.15 | 1.8 | 5.44 | 2.6 | 3.77  ±0.84 | 2.2  ±0.61 |

**Table S9 Peroxidase activity during maize development**

| **Tissue** | **Plastids** | | | | | | | |
| --- | --- | --- | --- | --- | --- | --- | --- | --- |
|  | **Assay 1** | | **Assay 2** | | **Assay 3** | | **Mean**  **mU/mL**  **± SE** | **Mean**  **Relative level**  **± SE** |
|  | mU/mL | Relative peroxidase activity | mU/mL | Relative peroxidase activity | mU/mL | Relative peroxidase activity |  |  |
| **L1** | 0.32 | 1.0 | 0.25 | 1.0 | 0.42 | 1.0 | 0.33  ±0.05 | 1.0  ±0.21 |
| **Stalk upper** | 0.58 | 1.8 | 0.38 | 1.6 | 0.72 | 1.7 | 0.56  ±0.10 | 1.7  ±0.40 |
| **Stalk lower** | 1.03 | 3.2 | 0.85 | 3.4 | 1.38 | 3.3 | 1.09  ± 0.15 | 3.3  ±0.68 |

| **Tissue** | **Mitochondria** | | | | | | | |
| --- | --- | --- | --- | --- | --- | --- | --- | --- |
|  | **Assay 1** | | **Assay 2** | | **Assay 3** | | **Mean**  **mU/mL**  **± SE** | **Mean**  **Relative level**  **± SE** |
|  | mU/mL | Relative peroxidase activity | mU/mL | Relative peroxidase activity | mU/mL | Relative peroxidase activity |  |  |
| **L1** | 0.22 | 1.0 | 0.14 | 1.0 | 0.26 | 1.0 | 0.21  ±0.03 | 1.0  ± 0.21 |
| **Stalk upper** | 0.24 | 1.1 | 0.18 | 1.3 | 0.34 | 1.3 | 0.26  ±0.05 | 1.2*  ± 0.30 |
| **Stalk lower** | 0.37 | 1.7 | 0.28 | 2.0 | 0.37 | 1.4 | 0.34  ±0.03 | 1.7*  ±0.28 |

**** Not significant at P value <0.05***

| **Tissue** | **Protoplasts** | | | | | | | |
| --- | --- | --- | --- | --- | --- | --- | --- | --- |
|  | **Assay 1** | | **Assay 2** | | **Assay 3** | | **Mean**  **mU/mL**  **± SE** | **Mean**  **Relative level**  **± SE** |
|  | mU/mL | Relative peroxidase activity | mU/mL | Relative peroxidase activity | mU/mL | Relative peroxidase activity |  |  |
| **Leaf** | 0.58 | 1.0 | 0.44 | 1.0 | 0.35 | 1.0 | 0.46  ±0.07 | 1.0  ±0.22 |
| **Stalk** | 0.78 | 1.4 | 0.67 | 1.5 | 0.65 | 1.9 | 0.70  ±0.04 | 1.6  ±0.25 |

**Table S10 Peroxidase activity under light and dark growth conditions**

| **Tissue** | **Plastids** | | | | | | | |
| --- | --- | --- | --- | --- | --- | --- | --- | --- |
|  | **Assay 1** | | **Assay 2** | | **Assay 3** | | **Mean**  **mU/mL**  **± SE** | **Mean**  **Relative level**  **± SE** |
|  | mU/mL | Relative peroxidase activity | mU/mL | Relative peroxidase activity | mU/mL | Relative peroxidase activity |  |  |
| **Light** | 0.55 | 1.0 | 0.39 | 1.0 | 0.49 | 1.0 | 0.48  ±0.05 | 1.0  ±0.15 |
| **Dark** | 1.47 | 2.7 | 1.16 | 3.0 | 1.39 | 2.8 | 1.34  ±0.09 | 2.8  ±0.35 |

| **Tissue** | **Mitochondria** | | | | | | | |
| --- | --- | --- | --- | --- | --- | --- | --- | --- |
|  | **Assay 1** | | **Assay 2** | | **Assay 3** | | **Mean**  **mU/mL**  **± SE** | **Mean**  **Relative level**  **± SE** |
|  | mU/mL | Relative peroxidase activity | mU/mL | Relative peroxidase activity | mU/mL | Relative peroxidase activity |  |  |
| **Light** | 0.38 | 1.0 | 0.44 | 1.0 | 0.61 | 1.0 | 0.48  ±0.07 | 1.0  ±0.21 |
| **Dark** | 0.99 | 2.6 | 0.69 | 1.5 | 1.13 | 1.9 | 0.94  ±0.12 | 2.0  ±0.38 |

| **Tissue** | **Protoplasts** | | | | | | | |
| --- | --- | --- | --- | --- | --- | --- | --- | --- |
|  | **Assay 1** | | **Assay 2** | | **Assay 3** | | **Mean**  **mU/mL**  **± SE** | **Mean**  **Relative level**  **± SE** |
|  | mU/mL | Relative peroxidase activity | mU/mL | Relative peroxidase activity | mU/mL | Relative peroxidase activity |  |  |
| **Light** | 0.77 | 1.0 | 0.61 | 1.0 | 0.58 | 1.0 | 0.65  ±0.06 | 1.0  ±0.13 |
| **Dark** | 1.76 | 2.3 | 1.64 | 2.7 | 1.14 | 2.0 | 1.51  ±0.20 | 2.3  ±0.37 |

**Table S11 Levels/activity of antioxidant agents during maize development**

**Catalase (CAT) activity**

| **Tissue** | **Protoplasts** | | | | | | | |
| --- | --- | --- | --- | --- | --- | --- | --- | --- |
|  | **Assay 1** | | **Assay 2** | | **Assay 3** | | **Mean**  **U/mL**  **± SE** | **Mean**  **Relative activity**  **± SE** |
|  | U/mL | Relative catalase activity | U/mL | Relative catalase activity | U/mL | Relative catalase activity |  |  |
| **Leaf** | 1.09 | 1.0 | 0.87 | 1.0 | 1.55 | 1.0 | 1.17  ±0.20 | 1.0  ±0.24 |
| **Stalk** | 3.06 | 2.8 | 2.52 | 2.9 | 3.94 | 2.5 | 3.17  ±0.40 | 2.7  ±0.57 |

**Total Glutathione (GSH) levels**

| **Tissue** | **Protoplasts** | | | | | | | |
| --- | --- | --- | --- | --- | --- | --- | --- | --- |
|  | **Assay 1** | | **Assay 2** | | **Assay 3** | | **Mean**  **µM**  **± SE** | **Mean**  **Relative level**  **± SE** |
|  | µM | Relative GSH levels | µM | Relative GSH levels | µM | Relative GSH levels |  |  |
| **Leaf** | 0.92 | 1.0 | 0.78 | 1.0 | 0.84 | 1.0 | 0.85  ±0.04 | 1.0  ±0.07 |
| **Stalk** | 1.88 | 2.0 | 1.75 | 2.2 | 1.93 | 2.3 | 1.85  ±0.05 | 2.2  ±0.12 |

**Ascorbic acid (AsA) levels**

| **Tissue** | **Protoplasts** | | | | | | | |
| --- | --- | --- | --- | --- | --- | --- | --- | --- |
|  | **Assay 1** | | **Assay 2** | | **Assay 3** | | **Mean**  **nM**  **± SE** | **Mean**  **Relative level**  **± SE** |
|  | nM | Relative AsA activity | nM | Relative AsA levels | nM | Relative AsA levels |  |  |
| **Leaf** | 1.27 | 1.0 | 1.21 | 1.0 | 1.29 | 1.0 | 1.26  ±0.02 | 1.0  ±0.02 |
| **Stalk** | 1.61 | 1.3 | 1.49 | 1.2 | 1.59 | 1.2 | 1.56  ±0.03 | 1.2  ±0.03 |

**Table S12 Levels/activity of antioxidant agents under light and dark growth condition**

**Catalase (CAT) activity**

| **Tissue** | **Protoplasts** | | | | | | | |
| --- | --- | --- | --- | --- | --- | --- | --- | --- |
|  | **Assay 1** | | **Assay 2** | | **Assay 3** | | **Mean**  **U/mL**  **± SE** | **Mean**  **Relative activity**  **± SE** |
|  | U/mL | Relative catalase activity | U/mL | Relative catalase activity | U/mL | Relative catalase activity |  |  |
| **Light** | 4.57 | 1.0 | 3.89 | 1.0 | 6.79 | 1.0 | 5.08  ±0.87 | 1.0  ±0.24 |
| **Dark** | 10.0 | 2.2 | 11.99 | 3.1 | 12.96 | 1.9 | 11.64  ±0.88 | 2.4  ±0.43 |

**Total Glutathione (GSH) levels**

| **Tissue** | **Protoplasts** | | | | | | | |
| --- | --- | --- | --- | --- | --- | --- | --- | --- |
|  | **Assay 1** | | **Assay 2** | | **Assay 3** | | **Mean**  **µM**  **± SE** | **Mean**  **Relative level**  **± SE** |
|  | µM | Relative GSH levels | µM | Relative GSH levels | µM | Relative GSH levels |  |  |
| **Light** | 1.26 | 1.0 | 1.18 | 1.0 | 1.24 | 1.0 | 1.23  ±0.02 | 1.0  ±0.02 |
| **Dark** | 3.46 | 2.7 | 3.48 | 2.9 | 3.38 | 2.7 | 3.44  ±0.03 | 2.8  ±0.05 |

**Ascorbic acid (AsA) levels**

| **Tissue** | **Protoplasts** | | | | | | | |
| --- | --- | --- | --- | --- | --- | --- | --- | --- |
|  | **Assay 1** | | **Assay 2** | | **Assay 3** | | **Mean**  **nM**  **± SE** | **Mean**  **Relative level**  **± SE** |
|  | nM | Relative AsA activity | nM | Relative AsA levels | nM | Relative AsA levels |  |  |
| **Light** | 2.21 | 1.0 | 2.19 | 1.0 | 2.31 | 1.0 | 2.24  ±0.04 | 1.0  ±0.03 |
| **Dark** | 3.16 | 1.4 | 2.94 | 1.3 | 3.12 | 1.4 | 3.07  ±0.07 | 1.4  ±0.04 |

**Table S13 8-OHdG level during maize development**

| **Tissue** | **Plastids** | | | | | | | |
| --- | --- | --- | --- | --- | --- | --- | --- | --- |
|  | **Assay 1** | | **Assay 2** | | **Assay 3** | | **Mean**  **ng/mL**  **± SE** | **Mean**  **Relative level**  **± SE** |
|  | ng/mL | Relative 8-OHdG level | ng/mL | Relative 8-OHdG level | ng/mL | Relative 8-OHdG level |  |  |
| **Stalk lower** | 1.31 | 1.0 | 1.38 | 1.0 | 1.13 | 1.0 | 1.27  ±0.07 | 1.0  ±0.08 |
| **Stalk upper** | 1.77 | 1.4 | 1.83 | 1.3 | 1.33 | 1.2 | 1.64  ±0.15 | 1.4*  ±0.14 |
| **L1** | 1.93 | 1.5 | 1.95 | 1.4 | 1.76 | 1.6 | 1.88  ±0.06 | 1.6*  ±0.09 |

**** Not significant at P value <0.05***

| **Tissue** | **Mitochondria** | | | | | | | |
| --- | --- | --- | --- | --- | --- | --- | --- | --- |
|  | **Assay 1** | | **Assay 2** | | **Assay 3** | | **Mean**  **ng/mL**  **± SE** | **Mean**  **Relative level**  **± SE** |
|  | ng/mL | Relative 8-OHdG level | ng/mL | Relative 8-OHdG level | ng/mL | Relative 8-OHdG level |  |  |
| **Stalk lower** | 1.50 | 1.0 | 1.49 | 1.0 | 1.40 | 1.0 | 1.46  ±0.03 | 1.0  ±0.03 |
| **Stalk upper** | 1.60 | 1.1 | 1.77 | 1.2 | 1.62 | 1.2 | 1.66  ±0.03 | 1.2*  ±0.04 |
| **L1** | 1.90 | 1.3 | 2.07 | 1.4 | 1.97 | 1.4 | 1.98  ±0.05 | 1.4*  ±0.04 |

**** Not significant at P value <0.05***

**Table S14 8-OHdG level under light and dark growth conditions**

| **Tissue** | **Plastids** | | | | | | | |
| --- | --- | --- | --- | --- | --- | --- | --- | --- |
|  | **Assay 1** | | **Assay 2** | | **Assay 3** | | **Mean**  **ng/mL**  **± SE** | **Mean**  **Relative level**  **± SE** |
|  | ng/mL | Relative 8-OHdG level | ng/mL | Relative 8-OHdG level | ng/mL | Relative 8-OHdG level |  |  |
| **Dark** | 0.46 | 1.0 | 0.43 | 1.0 | 0.51 | 1.0 | 0.47  ±0.02 | 1.0  ±0.06 |
| **Light** | 0.63 | 1.4 | 0.58 | 1.3 | 0.74 | 1.5 | 0.65  ±0.05 | 1.4  ±0.12 |

| **Tissue** | **Mitochondria** | | | | | | | |
| --- | --- | --- | --- | --- | --- | --- | --- | --- |
|  | **Assay 1** | | **Assay 2** | | **Assay 3** | | **Mean**  **ng/mL**  **± SE** | **Mean**  **Relative level**  **± SE** |
|  | ng/mL | Relative 8-OHdG level | ng/mL | Relative 8-OHdG level | ng/mL | Relative 8-OHdG level |  |  |
| **Dark** | 0.59 | 1.0 | 0.72 | 1.0 | 0.75 | 1.0 | 0.70  ±0.02 | 1.0  ±0.08 |
| **Light** | 0.86 | 1.5 | 0.94 | 1.3 | 0.95 | 1.3 | 0.92  ±0.04 | 1.4  ±0.08 |
